# Cellular and functional evaluation of LDLR missense variants reported in hypercholesterolemic patients demonstrates their hypomorphic impacts on trafficking and LDL internalization

**DOI:** 10.3389/fcell.2024.1412236

**Published:** 2024-07-24

**Authors:** Aseel A. Jawabri, Anne John, Mohammad A. Ghattas, Radwa E. Mahgoub, Mohammad I. K. Hamad, Maha T. Barakat, Bindu Shobi, Hinda Daggag, Bassam R. Ali

**Affiliations:** ^1^ Department of Genetics and Genomics, College of Medicine and Health Sciences, United Arab Emirates University, Abu Dhabi, United Arab Emirates; ^2^ College of Pharmacy, Al-Ain University, Abu Dhabi, United Arab Emirates; ^3^ Department of Anatomy, College of Medicine and Health Sciences, United Arab Emirates University, Abu Dhabi, United Arab Emirates; ^4^ Research Institute, Imperial College London Diabetes Centre (ICLDC), Abu Dhabi, United Arab Emirates; ^5^ ASPIRE Precision Medicine Research Institute Abu Dhabi, United Arab Emirates University, Al Ain, United Arab Emirates

**Keywords:** familial hypercholesterolemia (FH), low-density lipoprotein receptor (LDLR), low-density lipoprotein (LDL), receptor-mediated endocytosis, protein quality control, ER stress, ER associated protein degradation (ERAD)

## Abstract

**Background:**

Familial hypercholesterolemia (FH) is an autosomal dominant disorder characterized by increased LDL-cholesterol levels. About 85% of FH cases are caused by *LDLR* mutations encoding the low-density lipoprotein receptor (LDLR). LDLR is synthesized in the endoplasmic reticulum (ER) where it undergoes post-translational modifications and then transported through Golgi apparatus to the plasma membrane. Over 2900 LDLR variants have been reported in FH patients with limited information on the pathogenicity and functionality of many of them. This study aims to elucidate the cellular trafficking and functional implications of LDLR missense variants identified in suspected FH patients using biochemical and functional methods.

**Methods:**

We used HeLa, HEK293T, and LDLR-deficient-CHO-ldlA7 cells to evaluate the subcellular localization and LDL internalization of ten LDLR missense variants (p.C167F, p.D178N, p.C243Y, p.E277K, p.G314R, p.H327Y, p.D477N, p.D622G, p.R744Q, and p.R814Q) reported in multiethnic suspected FH patients. We also analyzed the functional impact of three variants (p.D445E, p.D482H, and p.C677F), two of which previously shown to be retained in the ER.

**Results:**

We show that p.D622G, p.D482H, and p.C667F are largely retained in the ER whereas p.R744Q is partially retained. The other variants were predominantly localized to the plasma membrane. LDL internalization assays in CHO-ldlA7 cells indicate that p.D482H, p.C243Y, p.D622G, and p.C667F have quantitatively lost their ability to internalize Dil-LDL with the others (p.C167F, p.D178N, p.G314R, p.H327Y, p.D445E, p.D477N, p.R744Q and p.R814Q) showing significant losses except for p.E277K which retained full activity. However, the LDL internalization assay is only to able evaluate the impact of the variants on LDL internalization and not the exact functional defects such as failure to bind LDL. The data represented illustrate the hypomorphism nature of variants causing FH which may explain some of the variable expressivity of FH.

**Conclusion:**

Our combinatorial approach of *in silico*, cellular, and functional analysis is a powerful strategy to determine pathogenicity and FH disease mechanisms which may provide opportunitites for novel therapeutic strategies.

## 1 Introduction

Familial Hypercholesterolemia (FH) (OMIM 143890) is a generally underdiagnosed and undertreated disorder affecting ∼1 in 250–300 individuals in most populations (Beheshti et al., 2020; Diboun et al., 2022; Cuchel et al., 2023; de Sá et al., 2023). The most common form of FH is inherited in an autosomal dominant manner and is caused by pathogenic haploinsufficiency loss-of-function variants in the low-density lipoprotein receptor (*LDLR*) gene, resulting in reduced clearance of low-density lipoprotein cholesterol particles (LDL-C) from the bloodstream. Mutations in LDLR account for ∼85% of FH cases with the remainder caused by either loss-of-function mutations in the LDLR’s ligand Apolipoprotein B (APOB) (∼5–10%) or by gain-of-function mutations in Proprotein Convertase Subtilisin/Kexin type 9 (*PCSK9*) (∼2%) (Nordestgaard et al., 2013; Zubielienė et al., 2022). In addition, a small percentage of FH cases are inherited in an autosomal recessive manner and caused by biallelic loss-of-function mutations in the Low-Density Lipoprotein Receptor Adaptor Protein-1 (*LDLRAP1*) gene (Henderson et al., 2016). However, in a systematic analysis carried out on eleven different populations, it was estimated that the overall prevalence of FH is ∼0.33% with the majority of hypercholesterolemia cases are presumed to be multifactorial caused by genetic predisposition and environmental factors such as diet and obesity or undiagnosed FH (Toft-Nielsen et al., 2022). Persistent elevation of LDL-C in the blood of hypercholesteremic patients causes accumulation of cholesterol in the arteries via potentially multiple mechanisms (Jay et al., 2015; Yu et al., 2016) that subsequently leads to premature coronary artery disease (CAD). For example, homozygous FH patients (HoFH) which affect one in 250,00-360,000 individuals, experience premature CAD within the first 2 decades of life which is much earlier than heterozygous FH patients (HeFH), who begin to express FH phenotypic characteristics after the first 2 decades of life (Marusic et al., 2020; Turgeon et al., 2016; M. J. Varghese, 2014).

Cardiovascular diseases are a leading global cause of death and account for >25% of all deaths in the United Arab Emirates (UAE) making it the leading cause of death in the country (Loney et al., 2013). In addition, the annual statistics from the Health Authority of the Emirate of Abu Dhabi (HAAD) indicate that cardiovascular disease has accounted for ∼35% of the deaths in the emirate for at least a decade (https://www.haad.ae/haad/tabid/1516/Default.aspx). Furthermore, in a recent study, it was estimated that the prevalence of CVD in the UAE is higher than the global average (Al-Shamsi et al., 2019). Despite the high prevalence of CVD and hypercholesterolemia in the UAE, the pathogenesis and underlying causes remained largely unknown. However, recently, Rimbert et al. (2022) recruited 229 patients with high LDL-C, performed customized targeted next-generation sequencing, and subsequently identified a number of missense, nonsense, and frameshift mutations in the *LDLR, APOB, PCSK9,* and *LDLRAP1* genes (Rimbert et al., 2022). Although they indicated that the prevalence of mutations among suspected FH patients in the UAE is low, they reported that 15 of these 229 patients can be genetically diagnosed with FH with ten of them harboring LDLR missense heterozygous variants, of which nine missense variants have been previously reported in other populations of different ethnicities except for p.C167F, which was reported for the first time (Rimbert et al., 2022).

LDLR is a transmembrane receptor that plays a critical role in regulating cholesterol homeostasis and LDL-C levels in the blood (Go and Mani, 2012). To facilitate the passage of cholesterol to the plasma membrane, LDL molecules act as cholesterol carriers which are captured by LDLR and internalized via receptor-mediated endocytosis. LDLR interacts with LDL through ApoB-100, a protein component of the LDL particles. ApoB-100 acts as a recognition signal and has a high affinity for LDLR forming the LDLR-LDL receptor-ligand complex to mediate the internalization of LDL particles (Martínez-Oliván et al., 2014). Due to differences in pH between the extracellular environment and early endosomes, LDL dissociates from LDLR in early endosomes and is then transported further to late endosomes and eventually to lysosomes where it gets degraded freeing cholesterol to be released and used for various cellular processes (Brown and Goldstein, 1979; Abifadel and Boileau, 2023). LDLR on the other hand, can be recycled back from early endosomes through the recycling endosomes to the plasma membrane for other cycles of LDL internalization.

Like other secretory and endomembrane proteins, nascent LDLR is initially synthesized in the ER as an immature protein with a molecular weight of ∼120 KDa and is post-translationally modified by N- and O-glycosylation in the ER and the Golgi apparatus increasing its apparent molecular weight from ∼120 KDa to ∼160 KDa (Omer, 2018). Mature LDLR is transported by vesicular transport from the Golgi to the plasma membrane to perform its function (Omer, 2018).

According to the Human Gene Mutation Database (HGMD; https://www.qiagen.com/), more than 2,900 LDLR variations ranging from missense, nonsense, frameshift, deletions, and insertions have been reported among suspected FH patients worldwide (Stenson et al., 2014). These variations are classified into six classes according to their effect on LDLR function (Schaefer et al., 2012), with class I variations causing loss or reduced LDLR synthesis. Class II are defective in their trafficking, in which the precursor LDLR is either not transported into the ER (class IIA) or is retained in the ER lumen and is unable to traffic out of the ER for further post-translational modifications in the Golgi apparatus (class IIB) (Schaefer et al., 2012). Class III in which the mutant LDLR matures and reaches the plasma membrane but loses its affinity for ApoB100 and consequently fails to recognize circulating LDL-C particles. Class IV in which the LDLR binds to LDL-C but the complex fails to cluster in clathrin-coated pits. Class V in which the LDLR-LDL-C complex is internalized but the LDLR fails to be recycled and is consequently degraded by lysosomes. In class VI, the LDLR releases LDL-C, and the LDLR is recycled back to the cell surface but fails to be properly reinserted back into the plasma membrane (Galicia-Garcia et al., 2020). Recently, a seventh class (class VII) has been proposed in which the ectodomain of the mature LDLR is cleaved by metalloproteinases (Alabi et al., 2021). Among the aforementioned functional pathogenic classes of LDLR, class II has been reported to be the most prevalent, contributing to ∼50% of all FH cases (Li et al., 2004). Alterations in LDLR trafficking result in complete or partial retention of LDLR mutants in the ER leading to their degradation by the Endoplasmic Reticulum Associated Degradation (ERAD) (Oommen et al., 2020) or slow trafficking to the Golgi apparatus (Hobbs et al., 1990). In eukaryotes, proteins that are targeted to the secretory pathway but fail to fold properly or assemble correctly with their multi-subunit partners are recognized, dislocated by ER chaperones and translocation systems, ubiquitinated, and delivered to the cytosolic 26S proteasome for degradation (Vembar and Brodsky, 2008). This mechanism has been reported as the pathogenesis mechanism underlying numerous human monogenic loss-of-function conditions including cystic fibrosis, emphysema, and many other conditions (Badawi, Varghese, et al., 2023; Y. Chen et al., 2005; Gariballa and Ali, 2020; Guerriero and Brodsky, 2012). In addition, disruption of components of this pathway has been shown to cause human diseases or lethality (Badawi, Mohamed, et al., 2023).

**FIGURE 1:**
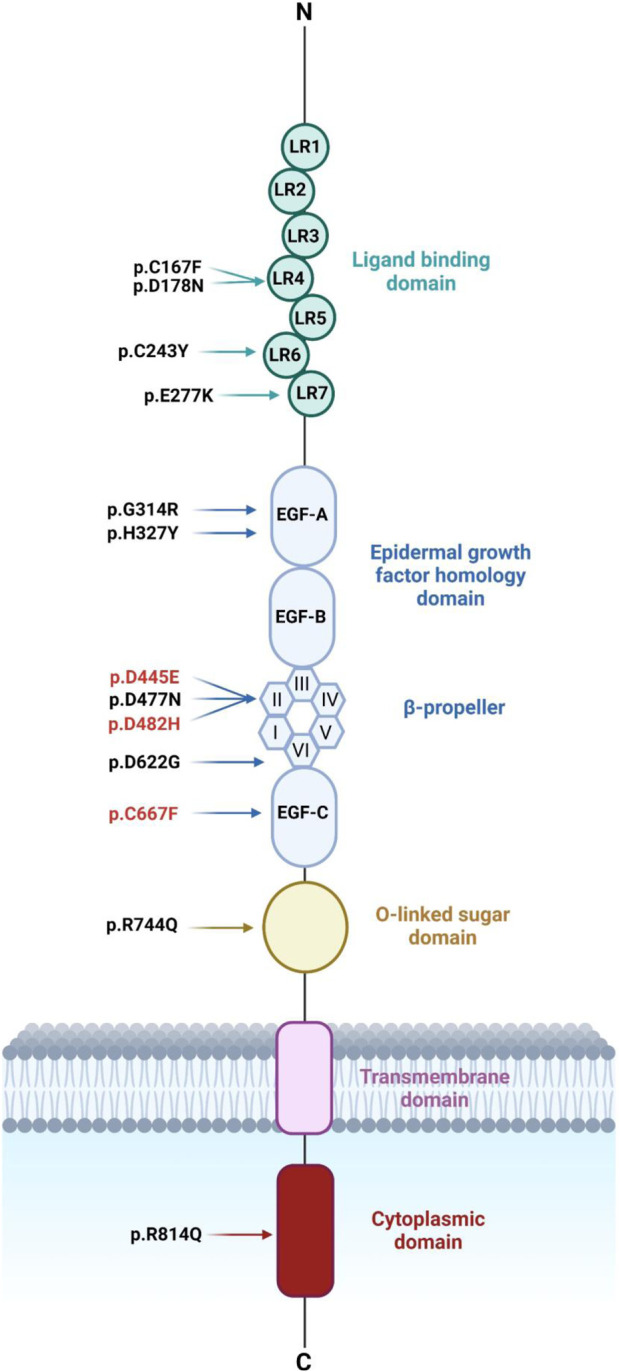
Schematic diagram representing LDLR’s structure with the ten LDLR missense variants identified recently in Emiratis (in black) and three other LDLR variants (in red). LDLR comprises 18 exons encoding five distinct domains each domain is represented by a different color. These domains include the ligand-binding domain (LBD) followed by the EGF homology domain consisting of EGF-A, EGF-B, β-propeller domain of six YWTD motifs, and EGF-C. The third domain is O-linked sugars followed by the transmembrane domain and the cytoplasmic domain. Figure 1 was generated using BioRender (https://app.biorender.com/). (Accessed on 20, June 2024). Biorender’s license is provided in Supplementary data sheet 3.

The main objective of this study is to determine the cellular localization and trafficking as well as their functional implications (specifically LDL internalization) of LDLR missense variants found in suspected FH patients (Rimbert et al., 2022). Most of these variants are found in Emiratis as well as patients from other populations and are located in multiple domains along the LDLR structure as shown in (Figure 1).

To achieve the main objectives, we first analyzed the potential impact of some of these variants by *in silico* analysis and molecular dynamics simulation followed by extensive experimental work including subcellular localization by immunofluorescence confocal microscopy, N-glycosylation profiles by Endoglycosidase H sensitivity and resistance assay and Western blotting analysis. Crucially, we carried out LDL internalization assays in CHO-ldlA7 cells (a well-established LDLR-knockout cell line) exogenously expressing WT LDLR or the studied variants to examine their impact on the functionality in terms of their ability to internalize LDL particles. This combinatorial approach revealed that the loss of LDLR function is variable and therefore a hypomorphic effect is evident for the majority of them. In addition, ER retention of some of the mutant proteins is a major (but not an exclusive) contributor to their loss of function.

## 2 Materials and methods

### 2.1 Pathogenicity prediction and *in silico* analysis

According to Rimbert et al. (2022), at least ten LDLR missense variants were found in Emirati patients suspected of having FH, many of which have been reported previously in patients from other populations. We re-examined their predicted pathogenicity using VEP (Ensembl Variant Effect Predictor; https://grch37.ensembl.org/info/docs/tools/vep/index.html) with the longest LDLR transcript (NM_000527.5) available on Ensemble (Martin et al., 2023). Each variant was classified based on score calculations and thresholds set by each prediction tool. The evaluation of VEP included seven prediction tools such as SIFT (Sorting Intolerant from Tolerant), PolyPhen-2 (Polymorphism Phenotype v 2), ClinPred Mutation Assessor, Mutation Taster, PROVEAN (Protein Variation Effect Analyzer), and clinical significance seen in Supplementary Table S1.

### 2.2 Molecular dynamic simulation analysis

The extracellular domain of the LDLR receptor was retrieved from the Protein Data Bank (PDB: 1N7D https://www.rcsb.org/) (Rudenko et al., 2002). The structure was prepared using MOE (Molecular Operating Environment, 2021), where all solvent atoms were removed from the structure, and any missing atoms, residues, chains, or loops were added using the protein preparation module. Protonate3D was then employed to assign each atom a unique protonation state. The mutated form of the structure was produced by mutating aspartic acid to glycine at position 622 and by mutating aspartic acid to histidine at position 482, using the Residue Scan module in MOE, producing the 3D structure of the mutant form. Both the wild type and mutated structures were then imported into Maestro (Madhavi Sastry et al., 2013), where the Protein Preparation Wizard (Madhavi Sastry et al., 2013) module is used to refine proteins to maintain structural integrity.

To gain a better understanding of how the mutation will affect the structure on a molecular level, MD simulation was performed on both the wild type and the mutated structures. Both structures were prepared and cleaned via the pdb4amber program, using the ff19SB force field to remove all water molecules. Both structures were then solvated by building a separate system for each using the XLeap in the AmberTools program, where the system was neutralized with Na + ions and solvated in an octahedral box of TIP3P water. The entire system was energy-minimized via the pmemd program in the AMBER 18 software package, while the solute atoms were restrained with a force constant of 500 kcal mol^−1^ Å^−2^. The entire system was then minimized without restrains for 1000 cycles. For the molecular dynamics simulation, the energy-minimized system was then gradually heated to the desired temperature of 300 K under NVT conditions. Using the Langevin thermostat, the SHAKE algorithm was employed for all bonds including hydrogen atoms with a collision frequency of 1.0 ps^−1^. Finally, the MD simulation was performed at 100 ns for the wild type, p.D482H and p.D622G at 300 K temperature and 1 atm pressure.

After completing the MD simulation, several analyses were performed on the obtained trajectories, including RMSD, RMSF, gyration, and hydrogen bond analysis. Unfortunately, since (1N7D) LDLR structure was only modeled for the extracellular domain, the calculated RMSD values for the wild type, p.D482H and p.D622G exceeded far beyond 3 Å. Therefore, to obtain more stable outputs for the wild type, the MD analysis was conducted on the LDLR sequence encoding the β-propeller region within the EGF-homology domain. According to (Jeon et al., 2001), 34% of the reported LDLR missense variants associated with FH were found in the YWTD repeats of the β propeller region within the EGF homology domain. Amongst the ten LDLR missense variants we studied, p.D482H and p.D622G were the only fully ER-retained variant located in repeat 2 and repeat 6 of the β propeller region respectively.

### 2.3 Generation of LDLR expression constructs

All the required LDLR missense variants were introduced by site-directed mutagenesis with *Pfu* Ultra HF polymerase (Stratagene, La Jolla, CA, United States) into the C-terminally HA-tagged pC3-LDLR plasmid (Kizhakkedath et al., 2019) using LDLR GenBank sequence (NM_000527). The mutagenic primers were designed using Primer X (bioinformatics.org/primerx) and are listed in Supplementary Table S2. Sanger DNA sequencing was performed using the dideoxy Sanger method by automated fluorescence sequencing on the ABI 3130xl Genetic Analyzer (Applied Biosystems, Waltham, MA, United States) to confirm the generation of intended amino acid changes within the LDLR cDNA.

### 2.4 Antibodies

For immunofluorescence, we used mouse monoclonal anti-HA-tag [(1:200; Cell Signaling Technologies (CST; Danvers, MA, United States)], rabbit polyclonal anti-calnexin (CANX 1:50; Santa Cruz Biotechnology, Dallas, TX, United States), Alexa Fluor 488-goat anti-rabbit IgG (1:200; Molecular Probes, Eugene, OR, United States), Alexa Fluor 555-goat anti-mouse IgG (1:200; Molecular Probes). For Western blotting, we used mouse monoclonal anti-HA [(1:4000; Cell Signaling Technologies (CST; Danvers, MA, United States)], mouse monoclonal anti-B actin (1:1000; Santa Cruz Biotechnology, Dallas, TX, United States).

### 2.5 Cell culture and transfection

For immunofluorescence, HeLa cells were cultured in Dulbecco’s modified Eagle’s Medium (DMEM; Invitrogen, Carlsbad, CA, United States) supplied with 10% fetal bovine serum (FBS; Invitrogen) and 100 U.ml^-1^ penicillin/streptomycin at 37°C with 5% CO_2_. Sterile coverslips were placed in a 24-well tissue culture plate and 1 mL of HeLa cells were added into each well and incubated for 24 h. HeLa cells were transiently transfected with 1.5 µL of Fugene HD transfection reagent (Promega, Madison, WI, United States), 1 µg of HA-tagged LDLR WT, or generated LDLR mutant constructs, and co-transfected with 0.5 µg GFP-HRas plasmid for 24 h. GFP-HRas was used as a plasma membrane marker.

For endoglycosidase H (Endo H) sensitivity and resistance assay and Western blotting analysis (described in subsequent sections), HEK293T cells (HEK-293T; ATCC, Manassas, VA, United States) were cultured in Dulbecco’s modified Eagle’s Medium (DMEM; Invitrogen, Carlsbad, CA, United States) supplemented with 10% fetal bovine serum (FBS; Invitrogen) and 100 U.ml^-1^ penicillin/streptomycin at 37°C with 5% CO_2_. HEK293T cells were transiently transfected with 3 µL of Fugene HD transfection reagent (Promega, Madison, WI, United States), 1 µg of HA-tagged LDLR WT, or generated LDLR mutant constructs in a 6-well tissue culture plate for 48 h.

The CHO-ldlA7 cells (Chinese hamster ovary cell line ldlA7, *LDLR* knockout cells; a generous gift from Dr. Monty Krieger, Massachusetts Institute of Technology, Cambridge, United States) were used for LDL internalization assay (described in the subsequent sections) and were cultured in Dulbecco’s Modified Eagle Medium/Nutrient Mixture F-12 (DMEM/F12; Invitrogen, Carlsbad, CA, United States) supplemented with 5% FBS (FBS; Invitrogen) and 100 U.ml^-1^ penicillin/streptomycin at 37°C with 5% CO_2_.

To block the proteasomal pathway, transiently transfected HEK293T cells with 1 µg LDLR WT or mutant expression constructs: p.D482H, p.D622G, and p.R744Q were serum starved for 4 h and treated with (10 µM) MG132 for 16 h.

### 2.6 Immunocytochemistry, confocal microscopy, and imaging

Twenty-four hours post-transfection; HeLa cells were fixed with ice-cold absolute methanol for 5 min at −20°C. Fixed cells were washed thrice with 1xPBS and blocked with 1% bovine serum albumin (BSA, Sigma) in 1xPBS. After blocking, HeLa cells were stained with mouse primary anti-HA antibody and rabbit anti-calnexin antibody, while HeLa cells co-transfected with GFP-HRas were incubated with mouse primary anti-HA antibody alone for 45 min at room temperature. The coverslips were then washed three times with 1xPBS, incubated with Alexa Fluor 555-goat anti-mouse and Alexa Fluor 488-goat anti-rabbit secondary antibodies for 45 min at room temperature, and mounted with immunofluor mounting medium (ICN biomedicals). Confocal microscopy and imaging were performed using the Nikon Eclipse system (Nikon Instruments Inc.) with FITC and TRITC filters. Images were captured with a ×100 oil immersion objective lens, adjusted, and merged using ImageJ (Fiji) software. All images shown are single sections in the z-plane.

To quantify the extent of colocalization between HA-tagged wildtype LDLR or LDLR missense variants and GFP-HRas/Calenxin, a colocalization analysis was performed using the colocalization threshold plugin on ImageJ (Fiji) software. Merged images were split into red (HA-tagged LDLR/variants) and green (GFP-HRas/Calnexin) channels and converted to 8-bit format for quantification. Individual cells were manually traced using the ROI manager plugin and analyzed in their corresponding channels. Comparative colocalization was determined by Pearsons correlation coefficient often denoted as R value ranging between (−1 to 1) where R = 1 positive correlation, R = 0 no correlation and R = −1 negative correlation between channels.

### 2.7 Western blot analysis and endoglycosidase H (Endo H) sensitivity and resistance assay

Twenty-four hours post-transfection, HEK293T cells were harvested with 1x PBS and centrifuged at 500 g for 5 min. Pellets were collected and lysed with a 1x protease inhibitor (Halt protease inhibitor cocktail; Thermo Fisher Scientific, Waltham, MA, United States). Total protein concentration was calculated using a bicinchoninic acid protein assay (BCA kit; Thermo Pierce). Protein samples were prepared with 5x Lamelli buffer and resolved in homemade 7.5% SDS/PAGE and blotted onto poly (vinylidene difluoride) membrane (Thermo Fisher Scientific). The PVDF membrane was probed with primary anti-HA antibody and anti-B actin antibodies overnight at 4°C, washed three times with 1xTBST, probed with secondary anti-mouse, washed three times with 1xTBST and developed using Enhanced Chemiluminescence Plus reagent (Thermo Pierce) and Typhoon FLA 9500 Imager (GE Healthcare Biosciences, Piscataway, NJ, United States). The relative abundance of LDLR protein variants was determined using densitometry analysis. ImageJ (Fiji) software was used to quantify band intensity by measuring the area of each protein band. The percentage of mature LDLR for each variant was calculated and a graph was generated on GraphPad Prism software (San Diego, CA, United States). One-way ANOVA was used to assess the significance of differences between LDLR variants *versus* the wild type as control. The Holm-Sidak method was utilized for statistical analysis, with *p*-values denoted as: (*) *p* ≤ 0.05; (**) *p* ≤ 0.01; (***) *p* ≤ 0.001. Data are represented as mean ± SEM from three independent experiments. Western blot replicates are indicated in Supplementary Figure S1.

For Endo H assay, a total of 60 µg of protein samples were prepared by adding 5X reaction buffer and denaturation buffer (2% SDS and 1M β-mercaptoethanol) added to each sample and boiled at 100°C for 5 min. After denaturation, each protein sample was divided into equal aliquots of 30 µg with or without 10U endoglycosidase H (Endo H; Sigma-Aldrich) and incubated at 37°C for 3.5 h. The samples were then loaded onto 7.5% SDS-PAGE gel for Western blot analysis as described above.

### 2.8 Dil-LDL internalization assay

For the Dil-LDL internalization time course experiment, CHO-ldlA7 cells were seeded onto sterilized coverslips in six wells of a 24-well plate. Seeded cells were transiently transfected with 100 ng of LDLR WT in each well using Lipofectamine 2000TM (Invitrogen, Burlington, Ontario, Canada) transfection reagent and incubated for 24 h. After transfection, cells were serum starved for 24 h using DMEM/F12 supplemented with 100 U.ml^-1^ penicillin/streptomycin only. After this 24-h serum starvation, Dil-LDL (Thermo Fisher Scientific, Waltham, MA, United States) was added to the cells at a concentration of 20 μg/ml prepared in serum-starved media, and the cells were incubated in the 37°C CO_2_ incubators for variable time points up to 4 h (i.e. 0, 20, 40, 60, 120 and 240 min). Then cells were fixed with freshly prepared 4% paraformaldehyde in PBS for 10 min at room temperature, washed three times with PBS, permeabilized with 0.2% TritonX-100 prepared in PBS for 10 min, washed three times with PBS and blocked in 0.1% BSA prepared in PBS at room temperature for 1 h. After blocking, cells were incubated with anti-HA primary antibody diluted in 0.1% BSA/PBS for 45 min at room temperature, washed three times with PBS, and then incubated with Alexa Fluor 488-goat anti-mouse secondary antibody diluted in 0.1% BSA/PBS for 45 min at room temperature. After secondary antibody incubation, the cells were washed three times with PBS. For nuclear staining, cells were incubated for 5 min with TO-PRO-3 iodide diluted in PBS and washed twice with PBS and once with distilled water. Finally, coverslips were mounted with an Immunofluor mounting medium (ICN biomedicals) and visualized by confocal laser scanning microscopy with FITC/TRITC filters.

After establishing that Dil-LDL internalization by the WT receptor was still in the linear range at 120 min, this time point was selected to test the internalization capacity of the mutants using the same protocol. Briefly, CHO-ldlA7 cells were transiently transfected with WT or the LDLR mutants individually using 100 ng of plasmids using Lipofectamine 2000TM transfection reagent.

Internalized Dil-LDL was quantified by tracing 30-50 cells individually in each image captured and measuring the mean values of total Dil-LDL signal intensity, area traced, and integrated density using ImageJ (Fiji) software following the same protocol used by (Potapova et al., 2011; McCloy et al., 2014; Bora et al., 2021). To counter any background signal, the background mean was also measured over the area that is the same for the measured cells. The integrated density obtained from the background was subtracted from the area of the cell and multiplied by the background mean, thus correcting it with the image background, giving us the corrected total cell fluorescence (CTCF) for each cell. Dil-LDL signal was measured for 30–50 LDLR wild-type transfected cells for each time point to establish the time-dependent internalization curve. A bar chart using GraphPad Prism software (San Diego, CA, United States) was plotted displaying individual data points shown in (Supplementary Figure S2).

For the subsequent mutant Dil-LDL internalization experiments, 90 transfected cells with the LDLR wild-type and a range of 20–80 transfected cells with the LDLR missense variants CTCF values were used. Each CTCF value was divided by the wild-type average and multiplied by 100 to convert to a percentage (%) relative to WT. The calculated CTCF values were plotted against the time of Dil-LDL internalization into a scatter plot using SigmaPlot software. A bar graph of (%) of Dil-LDL internalization was plotted for the LDLR wild-type and missense variants along with individual data points seen (Supplementary Figure S3) using GraphPad Prism software (San Diego, CA, United States).

## 3 Results

### 3.1 The p.D622G and p.R744Q variants seem to significantly affect LDLR trafficking to the plasma membrane

**FIGURE 2:**
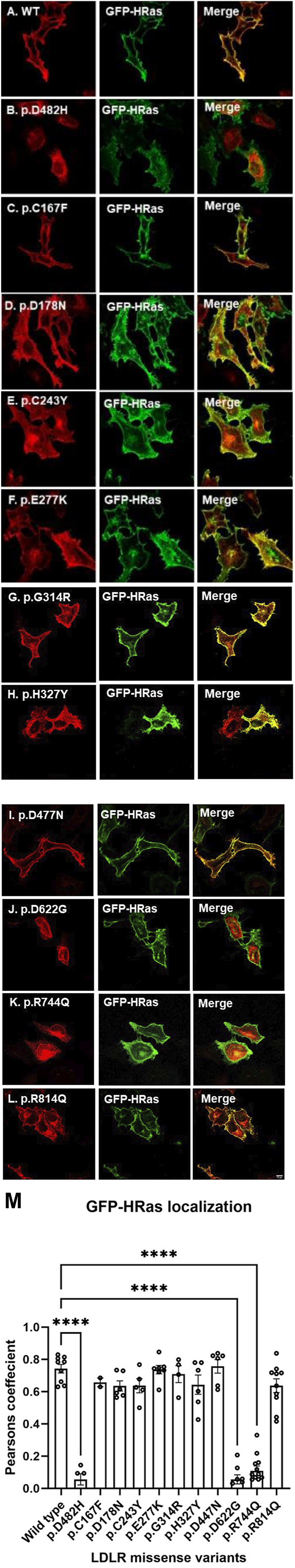
HeLa cells transiently transfected with HA-tagged LDLR wild-type [panel **(A)**] or mutant (p.D482H, p.C167F, p.D178N, p.C243Y, p.E277K, p.G314R, p.H327Y, p.D477N, p.D622G, p.R744Q and p.R814Q in panels [**(B–L)**, respectively] expression constructs were probed with anti-HA primary antibody and fluorescently stained in red with Alexa Fluor 555 as illustrated in the vertical panels **(A–L)** in the first and fourth columns. Cells were also co-transfected with GFP-tagged HRas plasmid acting as a plasma membrane marker as illustrated in vertical panels in the second and fifth columns. Merged images in vertical panels in the third and sixth columns demonstrate co-localization with GFP-tagged HRas. All images were captured using the Nikon Eclipse system (Nikon Instruments Inc., Tokyo, Japan) equipped with FITC and TRITC filters. Images were captured with a ×100 oil immersion objective lens. Images were enhanced and a scale bar was added using ImageJ (Fiji) software. Scale bar = 70 μm. Panel **(M)**: The degree of co-localization of GFP-HRas with LDLR wild type and missense variants. Data are presented as bar charts, with individual data points representing each analyzed cell. LDLR variants p.D482H, p.D622G, and p.R744Q exhibited significantly the lowest colocalization with GFP-HRas compared to LDLR wild type. The statistical significance of differences between LDLR variants and the wild type (used as control) was assessed using one-way ANOVA followed by the Holm-Sidak method. *p*-values are denoted as: () *p* ≤ 0.05; () *p* ≤ 0.01; () *p* ≤ 0.001.

We first investigated the subcellular localization of the ten newly generated LDLR missense variants by confocal immunofluorescence microscopy. HeLa cells were transiently transfected with expression constructs that either harbor the LDLR WT or the studied variants and co-transfected with GFP-tagged HRas plasmid, as a plasma membrane marker. The p.D482H mutant, previously reported by (Kizhakkedath et al., 2019) as a quantitatively ER-retained variant, was used as a positive control to evaluate ER retention. As expected, the LDLR wild-type seems to largely localize to the plasma membrane as evidenced by its co-localization with GFP-HRas shown in (Figure 2, panel A). The fraction that appeared to be intracellular is presumably the fraction of the receptor that is still in transit or has not yet matured. Eight of the ten LDLR missense variants tested (p.C167F, p.D178N, p.C243Y, p.E277K, p.G314R, p.H327Y, p.D477N, and p.R814Q) shown in Figure 2 (panels C, D, E, F, G, H, I and L, respectively) seemed to be largely localized to the plasma membrane, as indicated by their extensive co-localization with the plasma membrane marker GFP-HRas as shown in the merged images of each panel. However, variants p.D482H, p.D622G and p.R744Q (panels B, J and K, respectively) did not appear to localize to the plasma membrane or co-localize with GFP-HRas but instead exhibited perinuclear intracellular localization as shown in (Figure 2, panels B, J and K, respectively), suggesting that p.D622G and p.R744Q are largely ER-retained similarly to p.D482H as previously reported (Kizhakkedath et al., 2019; D. S. Varghese et al., 2023).

**FIGURE 3:**
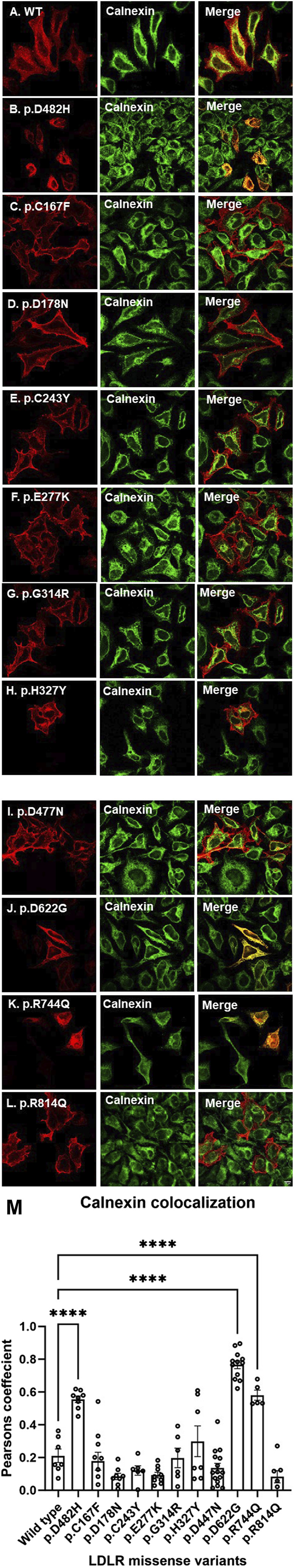
Transiently transfected HeLa cells expressing LDLR wild-type and missense variants (p.D482H, p.C167F, p.D178N, p.C243Y, p.E277K, p.G314R, p.H327Y, p.D477N, p.D622G, p.R744Q and p.R814Q in panels **(B–L)**, respectively probed with anti-HA antibody primary antibody and fluorescently stained with CST Alexa Fluor 555 secondary antibody as demonstrated in the first and fourth vertical panels **(A–L)**, which were also co-stained with ER marker calnexin probed with anti-calnexin primary antibody and fluorescently stained with Alexa Fluor 488 secondary antibody as demonstrated in the second and fifth vertical panels **(A–L)**. Images were merged to visualize co-localization with calnexin as displayed in vertical panels **(A–L)** in the third and sixth columns. All images were captured using the Nikon Eclipse system (Nikon Instruments Inc., Tokyo, Japan) equipped with FITC and TRITC filters. Images were captured with a ×100 oil immersion objective lens. Images intensity was enhanced in ImageJ in addition to the scale bar set at 70 μm. Panel **(M)**: The degree of co-localization of LDLR and missense variants with the ER marker, calnexin. Data are shown as bar charts, with individual data points representing each analyzed cell. LDLR variants p.D482H, p.D622G, and p.R744Q exhibited significantly the highest colocalization with GFP-HRas compared to LDLR wild type. The statistical significance of differences between LDLR variants and the wild type (used as control) was assessed using one-way ANOVA followed by the Holm-Sidak method. *p*-values are denoted as: () *p* ≤ 0.05; () *p* ≤ 0.01; () *p* ≤ 0.001.

Therefore, to further investigate the potential ER retention of p.D622G and p.R744Q, HeLa cells overexpressing LDLR missense variants were co-stained with calnexin, a well-established ER marker. As expected, p.D482H, co-localized with calnexin and p.D622G and p.R744Q seemed to largely co-localize with calnexin as well (Figure 3; panels J and K, respectively). On the other hand, p.C167F, p.D178N p.C243Y, p.E277Y, p.G314R, p.H327Y, p.D477N and R814Q did not colocalize strongly with calnexin as shown in (Figure 3 panels C, D, E, F, G, H, I and L, respectively). Therefore, these data strongly suggest that p.D622G and p.R744Q are largely ER-retained variants, while the others are largely localized to the plasma membrane.

The degree of co-localization was determined as described in the methods section. The bar charts were plotted on GraphPad Prism software (San Diego, CA, United States) representing the r values for LDLR wild type and missense variants, with individual data points representing each analyzed cell (minimum n = 5). Figure 2M represents the degree of colocalization of GFP-HRas with LDLR wild type and the missense variants. LDLR wild type showed the highest co-localization with GFP-HRas followed by p.D477N, p.E277K, p.G314R, p.C167F, p.R814Q, p.C243Y, p.D178N, and p.H327Y. As expected, p.D482H, p.D622G, and p.R744Q showed negligible colocalization with GFP-HRas compared to LDLR wild type. As for calnexin shown in Figure 3M which displays the degree of colocalization of LDLR WT and the missense variants with the ER marker, calnexin. As expected, p.D482H, p.D622G and p.R744Q displayed high degree of colocalization with calnexin compared to wild type while the other variants showed minimal (p.R814Q, p.E277K, and p.D178N) or variable degrees of colocalizations (p.D477N, p.C243Y, p.C167F, p.G314R, and p.H327Y). One-way ANOVA was used to assess the significance of differences between LDLR variants *versus* the wild type as control. The Holm-Sidak method was utilized for statistical analysis, with *p*-values denoted as: (*) *p* ≤ 0.05; (**) *p* ≤ 0.01; (***) *p* ≤ 0.001.

### 3.2 Western blotting and analyses of the N-glycosylation profiles further confirmed the ER retention of the p.D622G and p.R744Q variants

**FIGURE 4:**
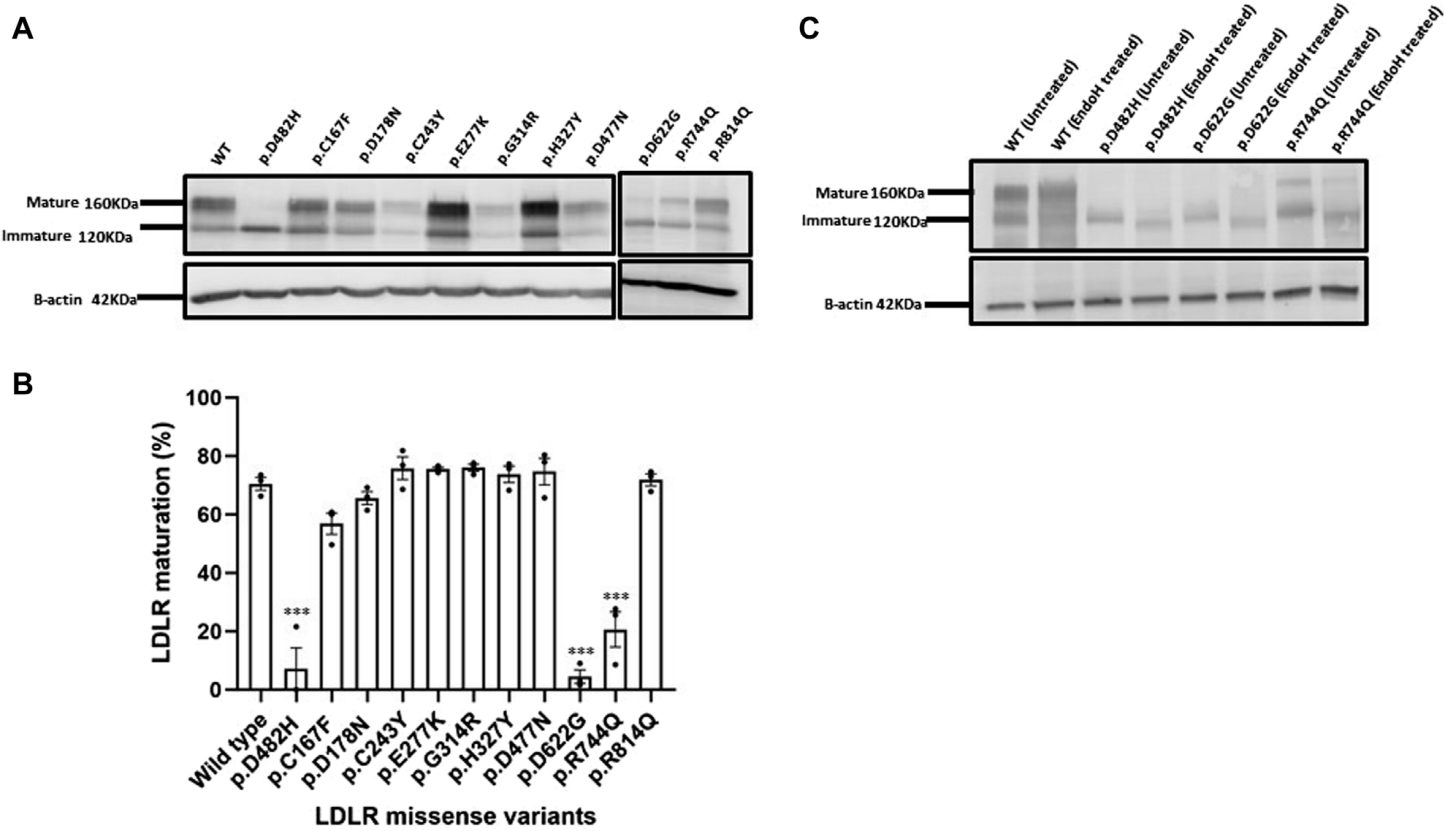
**(A)** HEK293T cells overexpressing HA-tagged LDLR or the indicated HA-tagged LDLR mutant constructs were transiently transfected for 48 h, harvested, quantified and probed with primary antibody against HA tag resolved on 7.5% SDS polyacrylamide gel for Western blot analysis. The immunoblot shows an upper band representing the mature LDLR form at ∼160 KDa and a lower band which is the immature form of LDLR at ∼120KDa. Β-actin was used as a loading control with an apparent MW of 42 KDa. **(B)** using GraphPad Prism software, a bar graph was created displaying the percentage (%) with individual data points of LDLR maturation of each LDLR missense variant relative to WT. Error bars represent ±SEM of three independent experiments; One-way ANOVA was used to calculate the statistical significance of LDLR variants *versus* the wild type. *p*-value was calculated using Holm-Sidak method which is represented as (*) *p* ≤ 0.05; (**) *p* ≤ 0.01; (***) *p* ≤ 0.001. **(C)** The glycosylation profiles of LDLR wild type and ER-retained missense variants: p.D482H (positive control), p.D622G and p.R744Q were examined by Endoglycosidase H enzyme assay. HEK293T cell lysates were divided into treated with EndoH and untreated groups, both incubated at 37°C for 3.5 h, probed against anti-HA primary antibody and analyzed by Western blot on a 7.5% polyacrylamide gel. Β-actin with an apparent MW of 42 KDa was used as a loading control.

To further confirm our suspicion from the confocal microscopy imaging, we analyzed the overexpression of the ten LDLR missense variants by Western blot analysis and Endo H resistance and sensitivity assays. As mentioned above, LDLR is initially synthesized as an immature form of LDLR with a molecular weight (MW) of ∼120 KDa and is later post-translationally modified upon passage through the secretory pathway to the mature form with an MW of ∼160 KDa. As expected (Figure 4A), the wild-type LDLR displayed two bands, an upper band at ∼160 KDa indicating the mature form of LDLR and a lower molecular weight band at ∼120 KDa indicating the precursor immature LDLR. Similar to the wild type, the exogenously expressed LDLR missense variants p.C167F, p.D178N p.C243Y, p.E277Y, p.G314R, p.H327Y, p.D477N, and p.R814Q displayed two bands as shown (Figure 4A) which were quantified in bar graph representing LDLR maturation (%) (Figure 4B). On the other hand, only the immature band was observed for p.D622G as seen in p.D482H variant as well, confirming its quantitative retention in the ER, while p.R744Q was partially retained in the ER with the majority of the protein in the immature form (Figure 4B). The results of the Western blots triplicates are shown in Supplementary Figure S1.

Based on the previous evidence and to further confirm ER retention, we focused on providing more experimental evidence on the maturation by testing the N-glycosylation profiles of the ER retained variants p.D482H, p.D622G and p.R744Q using the Endoglycosidase H sensitivity and resistance assay of their N-glycans. The enzyme Endo H cleaves off the immature N-glycans on nascent polypeptides that have not been transported from the ER to the Golgi apparatus where glycoproteins typically undergo further post-translational modifications. Further modeling of their N-glycans that renders them resistant to cleavage by Endo H. In (Figure 4C), on the immunoblot, we observed a shift in the MW of precursor LDLR (the lower molecular weight band) in Endo H treated samples: wild type, p.D482H, p.D622G and p.R744Q indicating susceptibility to Endo H, while the SDS-PAGE gel mobility of the mature form of LDLR wild-type remained unaffected, indicating resistance to Endo H treatment.

### 3.3 Molecular dynamics simulations reveal instability within the p.D482H and p.D622G variants

**FIGURE 5:**
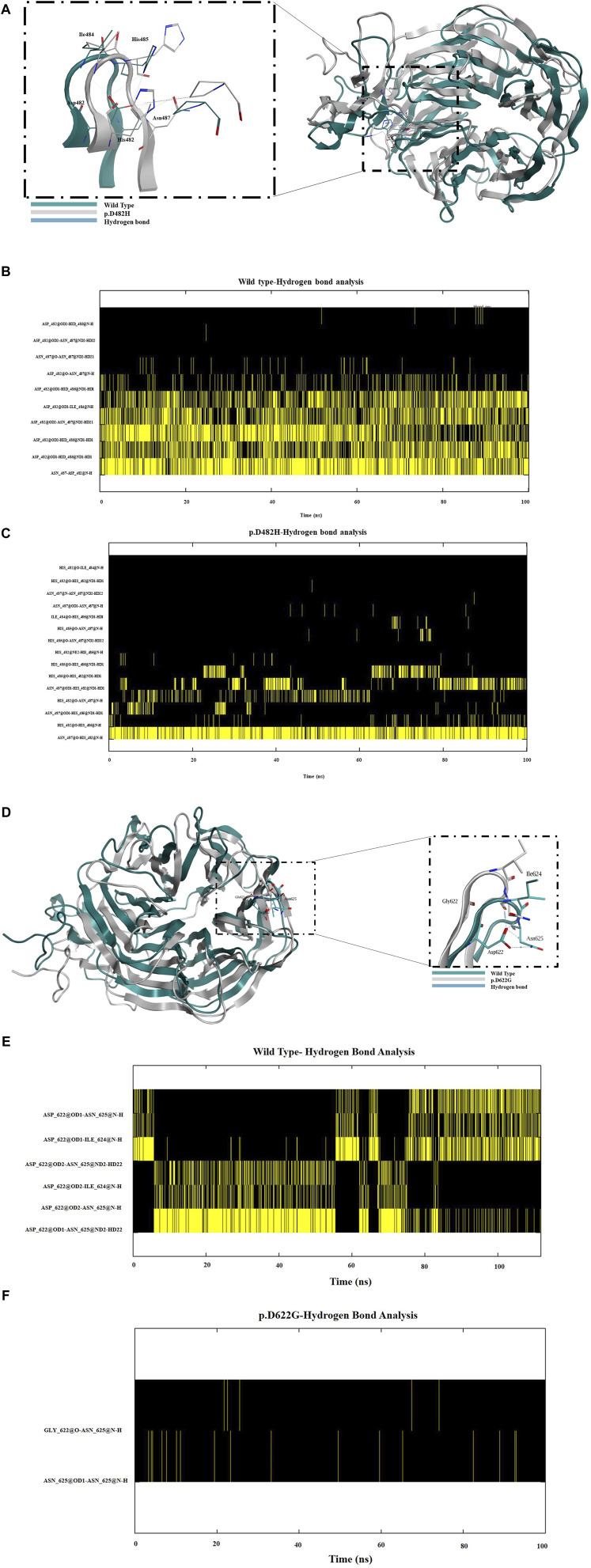
**(A)** A schematic diagram of the β propeller region in the EGF homology domain representing the wild type (in green), the LDLR missense variant p.D482H (in grey) and the hydrogen bonds established (in blue) between Asp482 with neighboring amino acids Ile484, His485 and Asn487. Similarly, **(D)** shows a schematic diagram of the β propeller region in the EGF homology domain representing the wild type (in green), the LDLR missense variant p.D622G (in grey) and the hydrogen bonds established (in blue) between Asp622 with neighboring amino acids Ile624 and Asn625. **(B)** Hydrogen bond analysis revealed that robust hydrogen bonds were formed between Asp482 and the neighboring amino acids Ile484, His485 and Asn487 but when substituted with His482 as seen in **(C)** very weak hydrogen bonds were formed with His485 and Asn487 and none with Ile484 but a strong hydrogen bond was formed between His482 and Asn487. (**E)** Hydrogen bond analysis revealed that Asp622 formed robust hydrogen bonds with amino acids Ile624 and Asn625. **(F)** Weak hydrogen bonds were formed between Asn625 and no hydrogen bonds were formed with Ile624.

Next, we assessed the stability and structural changes for p.D482H and p.D622G. Based on the results from the RMSD analysis in (Supplementary Figure S4A), which were run for 100 ns, the wild-type backbone structure exhibited limited deviations from its starting conformation, with RMSD values ranging between 1.3 Å and 1.6 Å. In contrast, p.D482H demonstrated a gradual increase in RMSD values from 0 ns to 80 ns, exceeding 3 Å at 80 ns, before slightly decreasing at 100 ns while the variant p.D622G showed a sudden deviation, particularly between the 10 ns and 20 ns intervals, reaching an RMSD value of almost 2 Å indicating instability. Based on the RMSD, RMSF was run for each amino acid residue to gain insight into the flexibility and mobility of the LDLR wild type, p.D482H, and p.D622G backbone structure. The RMSF calculations indicated far higher flexibility of p.D622G displaying multiple peaks at different amino acid positions with the highest peak exceeding 10 Å, compared to the wild type which showed more rigidity throughout the course of the simulation. However, p.D482H RMSF showed no difference at this timescale compared to the wild type as seen in (Supplementary Figure S4B). Afterward, gyration (Rg) was performed to evaluate the globularity and compactness of wild type, p.D482H and p.D622G. As depicted in (Supplementary Figure S4C), the LDLR wild type reaches a peak of 35 Å then converges at an average of 25 Å in comparison with p.D482H which initially diverges at 35 Å and slightly decreases to 33 Å at 100 ns and p.D622G which represented a sudden peak of 35 Å between 40 ns and 60 ns. These observations imply that p.D482H and p.D622G adopt less compact and folded mutant structures. Furthermore, subsequent examination of p.D482H and p.D622G structures using MOE software revealed that the Aspartic acid residue at position 482 forms strong hydrogen bonds with Ile484, His485, and Asn487 but when substituted with His482 it forms weaker hydrogen bonds with subsequent amino acids His485 and Asn487 and loses it is hydrogen bond with Ile484 as seen in (Figure 5A). As for p.D622G, Aspartic acid residue at position 622 forms hydrogen bonds with Ile624 and Asn625 but when substituted with Gly622 it forms no hydrogen bonds with subsequent amino acids as seen in (Figure 5D). Consequently, to confirm that, a hydrogen bond analysis was conducted on the two variants, focusing on Asp482 and Asp622 and their subsequent amino acids (Ile484, His485, and Asn487) and (Ile624 and Asn625) to assess the molecular interactions and stability of the wild type, p.D482H, and p.D622G variants. The analysis revealed that wild type Asp482 forms robust hydrogen bonds with Ile484, His485, and Asn487 and wild type Asp622 forms robust hydrogen bonds with Ile624 and Asn625 over 100 ns as seen in (Figures 5B,D). However, when substituted with His482, very weak hydrogen bonds were created with Asn487 and His485 while none were formed with Ile484. However, strong hydrogen bonds were created between His482 and Asn487 as seen in (Figure 5C) which were not observed in the wild type (Figure 5B). We predict that the new His482-Asn487 hydrogen bond formed could be the reason why p.D482H showed no difference in RMSD, RMSF, and Gyration compared to wild type. Moreover, no hydrogen bonds were formed between Gly at 622 with Ile624 and Asn625 as seen in (Figure 5F) which was not the case with wild type (Figure 5E). These alterations disrupt the structural integrity of LDLR and diminishes its stability. Overall, with reference to the data generated in RMSD, RMSF, radius of gyration (Rg), and hydrogen bond analysis we conclude that p.D482H and p.D622G are unstable.

### 3.4 The LDL internalization assessment indicates that p.C243Y, p.D445N, p.D482H, and p.D622G variants are severely dysfunctional while the other variants are partially dysfunctional, except for the p.E277K variant

**FIGURE 6:**
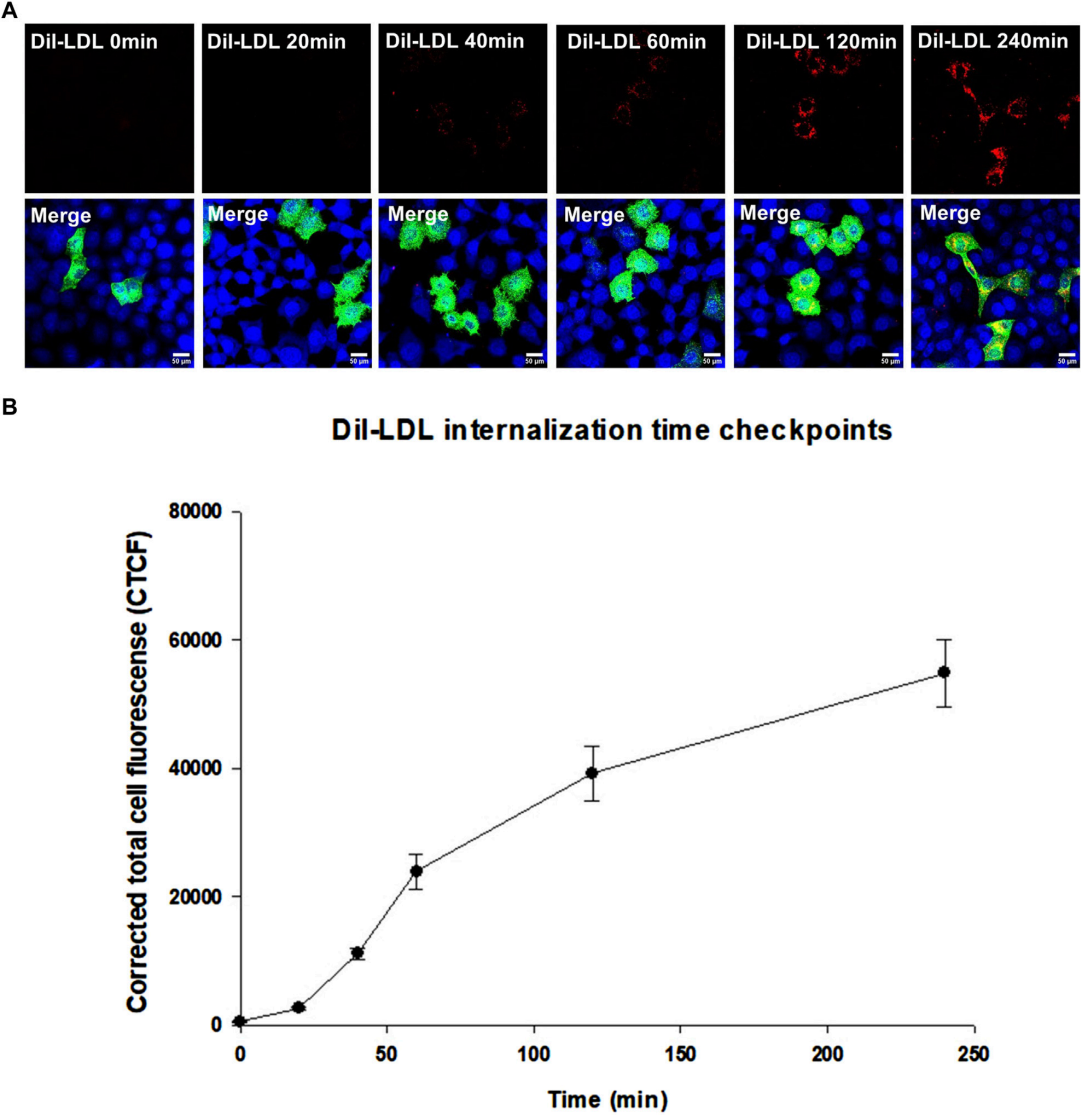
**(A)** represents Dil-LDL internalization by LDLR wild-type at six-time checkpoints (0 min, 20 min, 40 min, 60 min, 120 min and 240 min). All images were acquired using the Nikon Eclipse system (Nikon Instruments Inc., Tokyo, Japan) equipped with FITC and TRITC filters. Images were taken with a ×100 oil immersion objective lens. Images were enhanced and scale bar were added using ImageJ (Fiji) software. Scale bar = 50 μm. **(B)** is a scatterplot created using SigmaPlot 12.0 software showing a gradual increase in Dil-LDL internalization between 0 min and 60 min, becomes higher between 60 min and 120 min and stabilizing at 240 min. At least n = 30 of cells were used for quantification of Dil-LDL signal. Error bars represent ±SEM.

**FIGURE 7:**
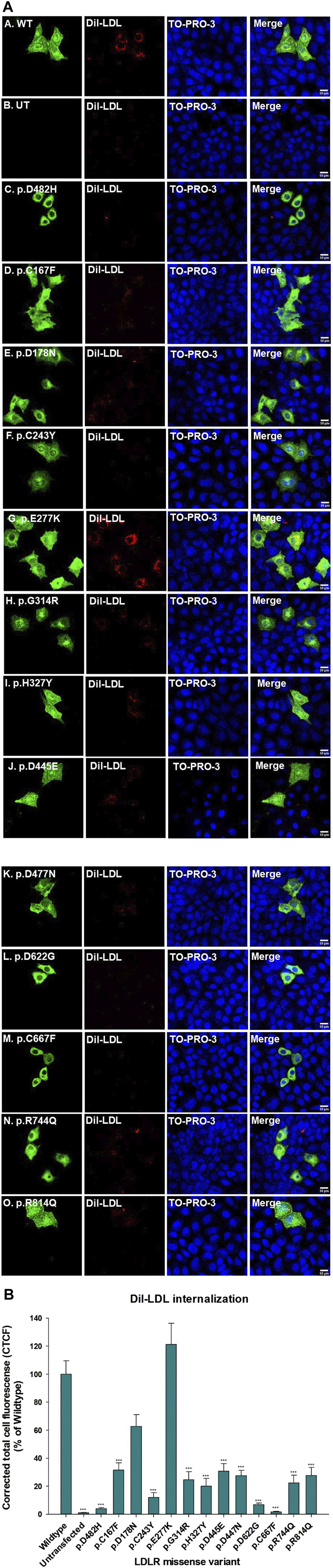
**(A)** CHO-ldlA7 (LDLR knockout) cells overexpressing HA-tagged LDLR wild-type or HA-tagged mutant expression constructs were treated with 20 μg/ml of Dil-LDL for 2 hours. The first and the fifth vertical panels **(a–o)** were probed with anti-HA primary antibody and stained with Alexa-Fluor 488. Dil-LDL is already fluorescently RFP tagged and is represented in the second and sixth vertical panels **(a–o)**. TO-PRO-3 iodide is a blue nucleus stain represented in the third and seventh vertical panels **(a–o)**. LDLR, Dil-LDL, and TO-PRO-3 iodide images were merged as seen in the fourth and eighth vertical panels **(a–o)**. All images were captured using the Nikon Eclipse system (Nikon Instruments Inc., Tokyo, Japan) equipped with FITC and TRITC filters in stacks. All images were merged using ImageJ (Fiji) software. Scale bar = 50 μm. **(B)** A bar graph was generated using SigmaPlot 12.0 software to measure the (%) of Dil-LDL internalization of all the LDLR missense variants in reference to the LDLR wild type. At least n = 20 cells were used to quantify Dil-LDL’s signal. p.E277K depicted the highest Dil-LDL internalization 20% higher than the wild type. p.D178N Dil-LDL internalization was reduced up to 60%. The rest of the LDLR missense variants have shown up to 60%–70% reduced Dil-LDL internalization. Statistical analysis was run with SigmaPlot 12.0 software using ANOVA on RANKS of the LDLR missense variants *versus* the wild type. The *p*-value was calculated at (*) *p* ≤ 0.05; (**) *p* ≤ 0.01; (***) *p* ≤ 0.001 using Dunn’s test.

Functional assays are the ultimate indicators for the impact of variants and we therefore wanted to evaluate the functional implications of the studied LDLR variants. To this end, we first performed a time course of Dil-LDL internalization by the exogenously expressed LDLR WT in a well-established LDLR-deficient cell line (CHO-ldlA7) (Kingsley and Krieger, 1984; Rodríguez-Jiménez et al., 2019; Rodríguez-Nóvoa et al., 2019). We found that Dil-LDL internalization is in a linear range for at least the first 120 min in cells expressing WT LDLR, whereas no significant internalization was observed in the untransfected CHO-ldlA7 cells. The time course (0 min, 20 min, 40 min, 60 min, 120 min, and 240 min) of internalization by CHO-ldlA7 cells expressing LDLR-WT is shown in (Figure 6A). We observed a visual gradual internal accumulation of Dil-LDL in the transfected cells which is not the case for the non-transfected cells (Figure 6A). We quantified the amount of internalized Dil-LDL for each time point as described in the methods section and then plotted the data against time as shown in Figure 6B with individual data points shown in Supplementary Figure S2. Based on this, the uptake of LDL appears to be linear within at least the first 2 hours and then begins to peak or slow down after 120 min. We therefore decided to examine the impact of the studied variants on Dil-LDL internalization at the 120 min time point as a suitable measure to indicate their functional implications. We tested all ten studied LDLR missense variants and three previously studied missense variants (p.D445E, p.D482H, and p.C667F) (Kizhakkedath et al., 2019). The variants were individually expressed in CHO-ldlA7 cells and Dil-LDL internalization was analyzed by laser confocal microscopy utilizing FITC/TRITC filters and representative cells of these mutants after 120 min of Dil-LDL internalization are shown in (Figure 7A). Obviously, as can be seen from the Dil-LDL signal in the middle panels, p.D482H, p.D622G, and p.C667F showed negligible Dil-LDL internalization, presumably due to their extensive ER retention, which classifies them as class II LDLR-FH-causing variants as shown in (Figure 7A panels C, L and M respectively). In addition, variants p.C167F, p.D178N, p.C243Y, p.G314R, p.H327Y, p.D445E, p.D477N, p.R744Q and p.R814Q have shown decreased Dil-LDL internalization compared to LDLR WT as shown in (Figure 7A panels D, E, F, H, I, J, K, N and O, respectively). Images of at least 50 transfected cells for each construct were quantified and plotted against WT as illustrated in Figure 7B with individual datapoints shown in Supplementary Figure S3. For example, p.D178N internalized 38% less Dil-LDL than WT while the other variants internalized 70%–80% less Dil-LDL than the wild type. Among the other variants was p.C243Y LDLR’s internalization was reduced by almost 85% compared to p.H327Y and p.R744Q Dil-LDL internalization which was reduced by 80% while p.C167F, p.G314R, p.D445E, p.D477N Dil-LDL internalization was reduced between 70% and 75%. An exception among the variants examined was the p.E277K variant, which showed extensive internalization similar to or even slightly higher than the WT by ∼15%.

### 3.5 LDLR missense variants p.D482H, p.D622G and p.R744Q are degraded via ERAD

To assess the degradation routes of the ER-retained variants, MG132 known to be a proteasomal inhibitor was used to treat transiently transfected HEK293T cells with wild type LDLR or the three ER-retained variants for 16 h. MG132 binds to the β5 subunit of the 20S proteasome inhibiting its proteolytic activity and as a result causes accumulation of ubiquitinated proteins (Bibo-Verdugo et al., 2017; Albornoz et al., 2019). The samples were run on 7.5% SDS-PAGE as described for Western blot analysis in the materials and methods section. ER-retained variants p.D482H, p.D622G and p.R744Q treated with MG132 were observed to have accumulated immature protein at 120 KDa compared to DMSO treated samples as seen on the representative immunoblot (Supplementary Figure S5A). The total mature and immature protein were calculated for each ER-retained variant and compared with transiently transfected HEK293T treated with DMSO (control). p.D482H (used as a positive control), p.D622G, and p.R744Q have shown a drastic increase in immature protein levels compared to their corresponding DMSO-treated samples. This indicates that all three ER-retained variants undergo proteasomal degradation through ERAD machinery (Supplementary Figures S5B, C). Densitometric analysis was performed for (n = 3) blots, normalized with β-actin, and plotted into two different bar graphs representing the percentage of total protein and the percentage of immature protein using GraphPad Prism software (San Diego, CA, United States).

## 4 Discussion

In this research article, we focused on evaluating the cellular, molecular, and biochemical impact of a series of LDLR missense variants found in Emirati and other patients suspected of having FH (Rimbert et al., 2022). The biochemical, molecular, and functional analysis of these LDLR missense variants helped to further clarify their pathogenicity as well as to elucidate their pathogenic mechanisms that may explain their contribution to the manifestation of FH. Throughout our study, we investigated the subcellular localizations, overexpression, intercellular folding and trafficking, molecular stability, and functionality of the ten LDLR missense variants found in Emiratis along with an additional three LDLR missense variants that were previously studied by performing functional analysis on them. Among the evaluated missense variants, only p.E277K did not show any impact of the trafficking or the function of LDLR and therefore could not be classified as a FH-LDLR-causing variant. The *in silico* analysis we conducted showed varying scores and predictions for other variants, but only p.E277K was correctly predicted as tolerant by most of the used prediction tools. In contrast, p.G314R and p.R744Q were mostly reported as tolerant or benign with conflicting levels of pathogenicity prediction. However, our experimental data revealed that p.G314R and p.R744Q are partially dysfunctional and therefore could be classified as class III and class IIB respectively. The only novel variant reported for the first time was p.C167F.

Among the ten LDLR variants, p.C167F, p.D178N, p.C243Y, and p.E277K are located in the ligand-binding domain (LBD), which consists of seven cysteine-rich ligand binding repeats (LRs) (Figure 1). Notably, the p.C167F variant disrupts a cysteine residue (C167) involved in a disulfide bridge with another cysteine (C184) within LR4. This disruption potentially affects the overall structure and stability of the LBD, hindering LDLR’s affinity for ApoB-100 in LDL and subsequently impairing its internalization through endocytosis (Figure 7A panel C; Figure 7B).

A 41-year-old Emirati FH patient with 9.4 mmol/L on lipid-lowering treatment (20 mg Simvastatin +10 mg Ezetimibe) was identified as a compound heterozygote carrying p.D178N and p.C243Y in LDLR (Rimbert et al., 2022). Interestingly, in another study, both p.D178N and p.C243Y were previously identified along with p.C167L in both alleles of the LDLR gene in a 13-year-old Pakistani male with HoFH who exhibited severe phenotypic characteristics of FH and was being treated with (40 mg Atorvastatin and 10 mg Ezetimibe) (Marusic et al., 2020). It is likely that, a substitution of Asp to Asn at position 178 would disrupt the conserved amino acid sequence Ser177-Asp178-Glu179 in the ligand binding domain, thereby reducing LDLR’s affinity for positively charged amino acids in ApoB molecules. This reduces LDL-C’s clearance as shown in (Figure 7A, panel D; Figure 7B).

In the case of p.C243Y, which was reported in a Chinese population study (D. Sun et al., 2018), we predict that C243Y, located at LR6, will presumably impact the formation of disulfide bridges between C243 and C261 in the ligand-binding domain, thereby affecting its stability and hindering its ability to clear LDL. Based on our experimental data, p.C243Y exhibited the lowest internalization of Dil-LDL among the non-ER retained variants. This lead us to speculate that LR6 is involved by interacting with LR2, LR3, and LR7 in the LBD during LDL binding, and a mutation in LR6 could explain the impaired binding activity of LDLR. Furthermore (Esser et al., 1988), explored the role of LRs by treating monkey COS cells with LDL composed of one ApoB100 ligand and β-VLDL composed of ApoB100 with other ApoE ligands. They found that LR1 had no affinity for either ligand, whereas LRs 2,3,6, and 7 achieved maximal binding of LDL. LR5 was found to bind to both ligands. In a recent report, LR4 and LR5 in the LBD together with the β-propeller domain influence the structural conformation of LDLR due to variations in pH and Ca^+^ levels destabilizing LR5 and converting LDLR from an open active form to a closed inactive form releasing bound LDL (Galicia-Garcia et al., 2020). Therefore, we conclude that p.C167F, p.D178N, and p.C243Y are class III: binding defective LDLRs.

The p.E277K variant was initially discovered in a Swedish family in 1995 by (Ulf Ekström et al., 1995) and was predicted to cause FH until (Pereira et al., 1995) identified it in a small Cuban family and sequenced the entire LDLR coding region, showing that this variant co-segregated within the family and affected three members with FH while two remained unaffected. Based on the results of (Pereira et al., 1995), (U. Ekström et al., 2000) decided to functionally characterize p.E277K alone by transfecting it into CHO-ldlA7 (LDLR-deficient) cells. According to functional data (U. Ekström et al., 2000), p.E277K did not influence LDL binding, uptake, or its degradation, which is consistent with our current data questioning its pathogenicity. However, when p.E277K was co-transfected with another variant, I402T in exon nine of the LDLR, LDL clearance was greatly reduced. This variant has been widely identified in European, South Asian, South African, Turkish and Mexican populations (Khoo et al., 2000; Mozas et al., 2000; Weiss et al., 2000; Fouchier et al., 2001; Sözen et al., 2005; Vaca et al., 2011; Bertolini et al., 2013; Van Zyl et al., 2014) and was recently functionally evaluated by (Rodríguez-Jiménez et al., 2019). Taken together, we conclude that p.E277K does not cause FH but it could cause FH in the presence of another variant (U. Ekström et al., 2000; Rodríguez-Jiménez et al., 2019).

Moreover, p.G314R and p.D477N were categorized into class III variants as well. p.G314 is the last amino acid found in exon 6, which encodes EGF-A. Previously, p.G314R was visualized by (Hurst et al., 2009; Usifo et al., 2012) in the Single Amino Acid Polymorphism Database (SAAPdb) and was classified as “uncertain pathogenicity”. We speculate, that a substitution to Arg, a positively charged amino acid, may repel Ca^2+^ ions required in providing stability for LR5 and the dissociation of LDL from LDLR in the endosome (Arias-Moreno et al., 2008). As a result, the binding affinity of the LDLR to LDL is reduced, making it unable to remove LDL. On the other hand, p.D477N was identified in a 45-year-old male Emirati carrying another different variant in the *PCSK9* gene with LDL-C of 18.2 mmol/L on lipid-lowering treatment (20 mg Rosuvastatin +10 mg Ezetimibe) (Rimbert et al., 2022). p.D477N was also identified in a 35-year-old man who died from Thrombosis of the coronary artery and was also reported among the list of LDLR missense variants found in the Saudi population affected by FH (Morad et al., 2018). They analyzed the structural impact of each missense variant and their binding affinities towards ApoB were tested by conducting molecular modeling and docking analysis (Morad et al., 2018). For their analysis, they selected the entire sequence of LDLR (860 amino acids) and selected only (400 amino acids) coding for LDLR’s binding site in ApoB. By comparing docking scores, they concluded that p.D477N was likely the cause of FH in Saudi patients, despite the lack of functional evidence (Morad et al., 2018). In agreement with their findings, we infer that p.D477N is binding defective LDLR and is FH-causing.

According to an MD simulation analysis by (Hyock et al., 2008), it was revealed that p.H327Y results in the upregulation of PCSK9 and formation of a hydrogen bond between p.H327Y in LDLR and Asp374 in PCSK9, consequently strengthening the affinity between LDLR and PCSK9, restricting the dissociation of LDLR from LDL and inhibiting LDLR from being recycled back to the plasma membrane (Jelassi et al., 2009). These observations are consistent with our functional evidence, suggesting that p.H327Y could potentially be classified as a class V: recycling defective LDLR.

The P.D622G variant was identified in a Czech population (Tichý et al., 2012) and was later functionally studied by (Galicia-Garcia et al., 2020). It was observed that a substitution from D622 to a G alters the trafficking of LDLR as illustrated by our localization, Western blot, and Endo H sensitivity assay. This is likely due to the fact that a substitution from D622 to G causes LDLR to become unstable and unable to form essential hydrogen bonds with adjacent amino acids but rather induces the formation of unfavorable hydrogen bonds with different amino acids as shown by our MD analysis (Figures 5A–C,E), leading to a complete dysfunction (Figure 7A, panel L; Figure 7B). Therefore, we classify p.D622G as a class IIA: trafficking deficient LDLR.

In addition, as a continuation of the analysis (Kizhakkedath et al., 2019; D. S. Varghese et al., 2023), we evaluated the functional impact of only p.D445E, p.D482H, and p.C667F located in the EGF homology domain (Figure 1), where p.D482H and p.C667F were classified as class IIA, while p.D445E was classified as class V: recycling defective LDLR, where p.D482H and p.C667F exhibited residual Dil-LDL internalization, whereas p.D445E internalized 35% Dil-LDL (Figure 7B). The EGF homology domain is composed of conserved cysteines that play a crucial role in LDLR stability and, when mutated, cause ER retention, which is the case for p.C667F, which is unable to form a disulfide bridge with C681 and C696 (Südhof et al., 1985; Kizhakkedath et al., 2019). The p.D445E and p.D482H variants are located in repeat two in the β-propeller region, which consists of conserved Asp residues and a YWTD motif stabilizing LDLR’s structure. We predict that mutations in this region destabilize the β propeller’s structure and obstruct the transition of LDLR from a closed form to an open form during LDL release in the endosome (Kizhakkedath et al., 2019; Galicia-Garcia et al., 2020).

In addition, p.R744Q was initially identified in an English patient with possible HeFH (X. M. Sun et al., 1997). Variants in the O-linked sugar domain were thought to have an insignificant impact on LDLR’s function and were unlikely to be the cause of FH (X. M. Sun et al., 1997). However, our findings suggest otherwise. We observed that p.R744Q co-localized with ER marker calnexin, but not with the plasma membrane marker GFP-HRas. This led us to speculate that p.R744Q may be retained in the ER which we further confirmed through Western blot and endoglycosidase H sensitivity assay to be partially ER retained and by internalization assay to be partially dysfunctional. Based on these results, we believe that p.R744Q belongs to class IIB: transport defective LDLR.

The mutation p.R814Q was found in exon 17, which encodes the cytoplasmic tail of the LDL receptor (LDLR). The amino acid R814 is conserved in six species and is crucial for LDLR activity. This residue is implicated in the clustering of clathrin-coated pits during the endocytosis of the LDLR-LDL complex, classifying p.R814Q as a class IV: internalization defective LDLR (Arca and Jokinen, 1998). p.R814Q was initially identified in a postmenopausal woman who participated in an *in vivo* study of LDL metabolism. Her results indicated a significant decrease in LDLR activity and elevated LDL levels (Arca and Jokinen, 1998). Consistent with our findings, p.R814Q was localized to the plasma membrane and expressed both mature and immature forms of LDLR. However, it exhibited significantly lower Dil-LDL internalization compared to the wild type. In an attempt to classify the classes of those variants, variants p.D482H, p.C667F, and p.D622G could be classified as class IIA (ER retained), and p.R744Q as class IIB (partially ER retained). Five of the variants (p.C167F, p.D178N, p.C243Y, p.G314R, and p.D477N) could also potentially be classified into class III (binding defective LDLR mutants) due to their possible loss of affinity to ApoB binding site located on LDL molecules. P.R814Q might be classified under class IV (internalization defective LDLR). Lastly, p.H327Y and p.D445E could be classified into class V as recycling defective LDLR.

We propose that the substitution of arginine to glutamine at position 814 disrupts the recruitment of clathrin by adaptor polypeptide 2 (AP2), inhibiting the formation of early endosomes by Bin/Amphiphysin/Rvs (BAR) domain proteins and Rab5 protein (Wandinger-Ness and Zerial, 2014) (Siddiqui et al., 2022), thereby impeding receptor-mediated endocytosis.

While this study identified the functional implications of LDLR-FH causing variants, in addition to LDLR, CD36 and Low-Density Lipoprotein receptor related protein-1 (LRP1) are multifunctional scavenger receptors that can bind to LDL but with lesser efficiency and rather have higher affinities to other forms of LDL (Jay et al., 2015; Yu et al., 2016). Loss-of-function in LDLR leads to elevated levels of uncleared LDL from the bloodstream. As a result, LDL gets oxidized and becomes a target to CD36 receptor in macrophages (Yu et al., 2016). CD36 internalizes oxidized LDL (Ox-LDL) mediating the formation of foam cells in macrophages increasing the risk of atherosclerosis. LRP-1 internalizes various ligands, including ApoE-rich lipoproteins and aggregated LDL (agLDL) (Potere et al., 2019). While gene therapy and natural LRP-1 agonists are potential therapeutic targets, further research is needed. Certain antibodies targeting LRP-1’s CR9 domain have been proposed to prevent agLDL uptake and foam cell formation, suggesting LRP-1 as a potential target for atherosclerosis treatment (J. Chen et al., 2021).

Moreover, other future potential therapeutic could be achieved by targeting the degradation routes of ER-retained variants through ERAD. Precursor LDLR is composed of a signal peptide that gets cleaved off once it enters the ER via the signal peptide complex (SPC) through a translocon to begin protein folding (Qi et al., 2017). Misfolded proteins activate the Endoplasmic-Reticulum Quality Control system (ERQC) where global ER-chaperons such as BiP, protein disulfide isomerases (ERdj family), calnexin and calreticulin are recruited for protein folding. Class IIA and B variants such as p.D482H p.D622G and p.R744Q undergo prolonged interactions with ERQC global chaperons and are delivered into the ERAD complex for ubiquitin-proteasomal degradation (Oommen et al., 2020). Inhibiting ubiquitin-proteasomal degradation route for FH-LDLR class II variants could rescue these variants and allow them to escape ER to the Golgi apparatus and then to the plasma membrane to perform their function.

## 5 Conclusion

In summary, we have highlighted the cellular and functional impact of the ten LDLR missense variants reported in Emirati and other suspected FH patients and classified nine as LDLR-FH-causing variants. The degree of loss of function in terms of LDL internalization among the studied variants has been shown to be variable and therefore demonstrating possible hypomorphic effects. Perhaps, this hypomorphic impact should be considered when interpreting variations in LDLR and possibly other genes (Badawi, Varghese, et al., 2023; Graça et al., 2023). The main clinical implications of these findings are their utility in confirming diagnosis due to their clear loss-of LDL internalization function that we demonstrated in this manuscript. In addition, they could also be used for cascade and population screening programmes to identify other affected individuals which can be used for early detection to implement preventative measures to reduce their pathological impact. We believe that our findings could also help in the development of future therapeutics targeting the trafficking pathways of the examined LDLR missense variants and treating patients with FH.

## Data Availability

All the required LDLR missense variants were introduced by site-directed mutagenesis with Pfu Ultra HF polymerase (Stratagene, La Jolla, CA, United States) into the C-terminally HA-tagged pC3-LDLR plasmid (Kizhakkedath et al., 2019) using LDLR GenBank transcript accession number: (NM_000527.5) available on (https://www.ncbi.nlm.nih.gov/nuccore/NM_000527.5/).
