# Supplementary material for: Cellular and functional evaluation of LDLR missense variants reported in hypercholesterolemic patients demonstrates their hypomorphic impacts on trafficking and LDL internalization
Source: Front Cell Dev Biol. 2024 Jul 24;12:1412236. doi: 10.3389/fcell.2024.1412236 (PMC11303217; doi:10.3389/fcell.2024.1412236)
Supplement: Supplementary file 1 [file DataSheet2.PDF]

Table 2: List of primers designed on PrimerX for the listed LDLR primers

LDLR missense variant	Primers
**p.C167F**	Forward: 5' CAGCTGTGGGCCTTCGACAACGACCC 3'<br>Reverse: 5' GGGTCGTTGTCGAAGGCCCACAGCTG 3'
**p.D178N**	Forward: 5' GAAGATGGCTCGAATGAGTGGCCGC 3'<br>Reverse: 5' GCGGCCACTCATTCGAGCCATCTTC 3'
**p.C243Y**	Forward: 5' CTGACGAATTCCAGTACTCTGATGGAAACTG 3'<br>Reverse: 5' CAGTTTCCATCAGAGTACTGGAATTCGTCAG 3'
**p.E277K**	Forward: 5' GTTAATGTGACACTCTGCAAGGGACCCAACAAGTTC 3'<br>Reverse: 5' GAACTTGTTGGGTCCCTTGCAGAGTGTCACATTAAC 3'
**p.G314R**	Forward: 5' CCATCAAAGAGTGCAGGACCAACGAATGC 3'<br>Reverse: 5' GCATTCGTTGGTCCTGCACTCTTTGATGG 3'
**p.H327Y**	Forward: 5' GGCGGCTGTTCCTACGTCTGCAATG 3'<br>Reverse: 5' CATTGCAGACGTAGGAACAGCCGCC 3'
**p.D477N**	Forward: 5' CATCCAGGCCCCCAACGGGCTGGCTG 3'<br>Reverse: 5' CAGCCAGCCCGTTGGGGGCCTGGATG 3'
**p.D622G**	Forward: 5' CAAAGTATTTTGGACAGGTATCATCAACGAAGCC 3‘<br>Reverse: 5' GGCTTCGTTGATGATACCTGTCCAAAATACTTTG 3'
**p.R744Q**	Forward: 5' CACACAACCACCCAACCTGTTCCCGAC 3'<br>Reverse: 5' GTCGGGAACAGGTTGGGTGGTTGTGTG 3'
**p.R814Q**	Forward: 5' CTATGGAAGAACTGGCAGCTTAAGAACATCAAC 3‘<br>Reverse: 5' GTTGATGTTCTTAAGCTGCCAGTTCTTCCATAG 3'
