# Supplementary figures and images for: Cellular and functional evaluation of LDLR missense variants reported in hypercholesterolemic patients demonstrates their hypomorphic impacts on trafficking and LDL internalization
Source: Front Cell Dev Biol. 2024 Jul 24;12:1412236. doi: 10.3389/fcell.2024.1412236 (PMC11303217; doi:10.3389/fcell.2024.1412236)

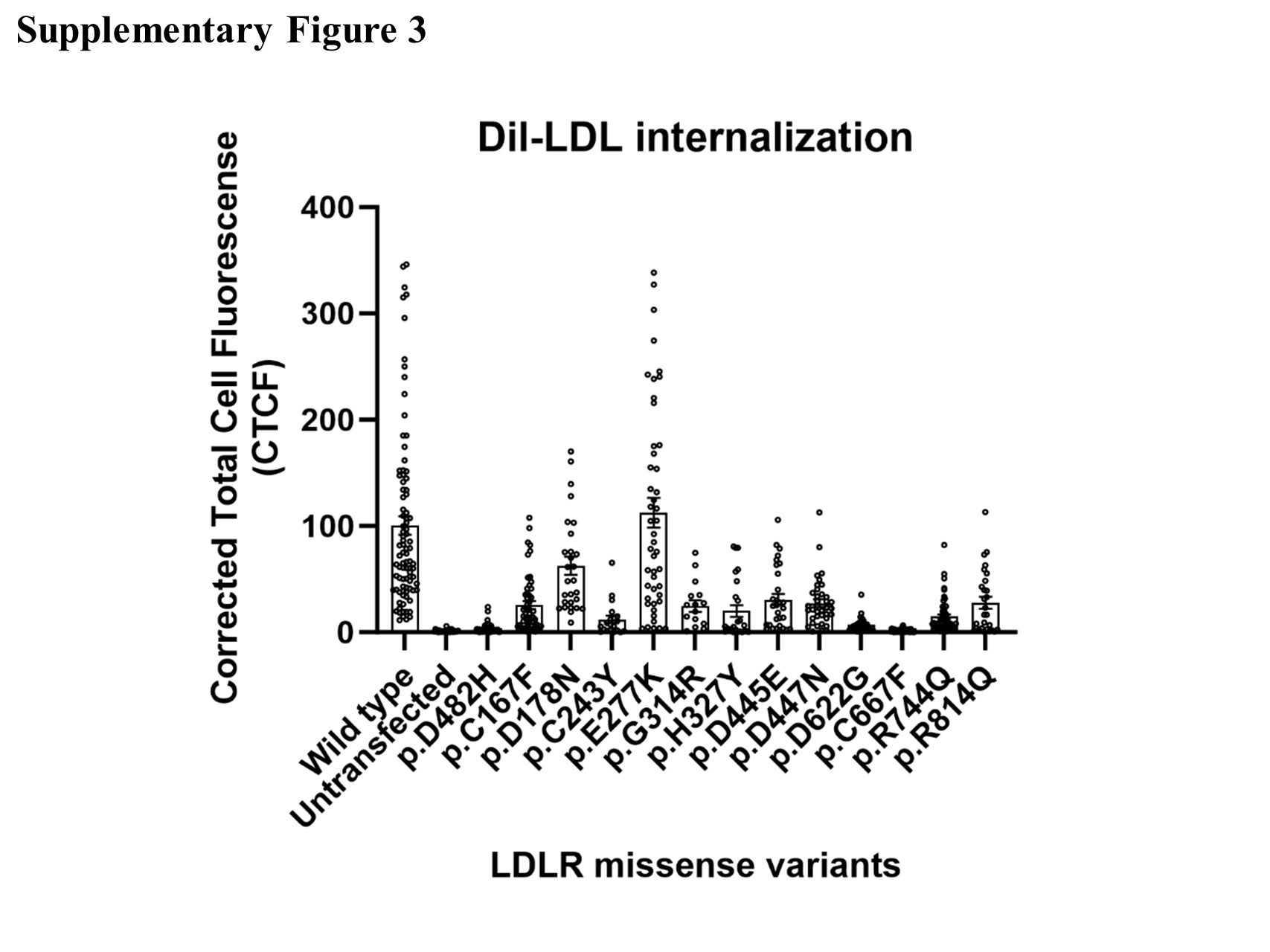

Supplement: Supplementary file 2 [file Image3.JPEG]

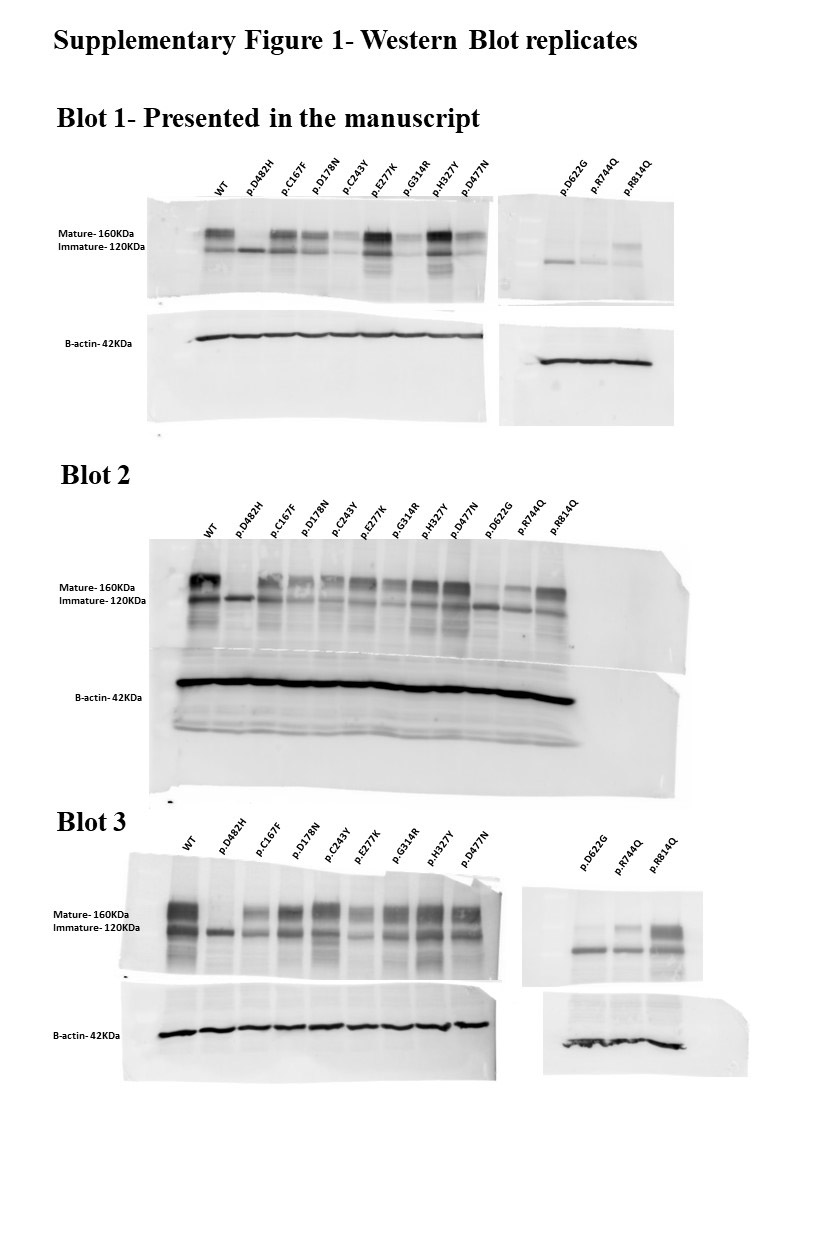

Supplement: Supplementary file 3 [file Image1.JPEG]

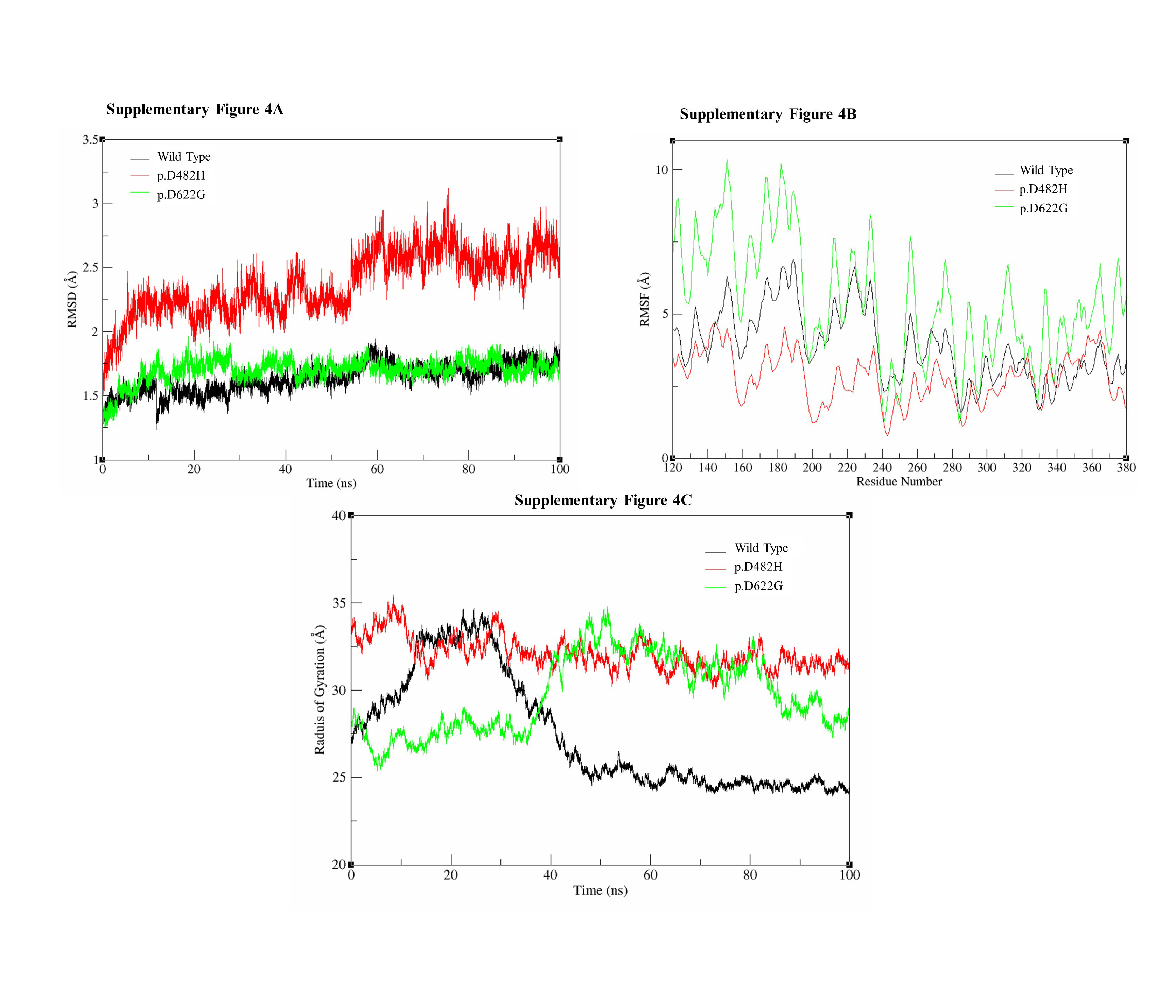

Supplement: Supplementary file 4 [file Image4.JPEG]

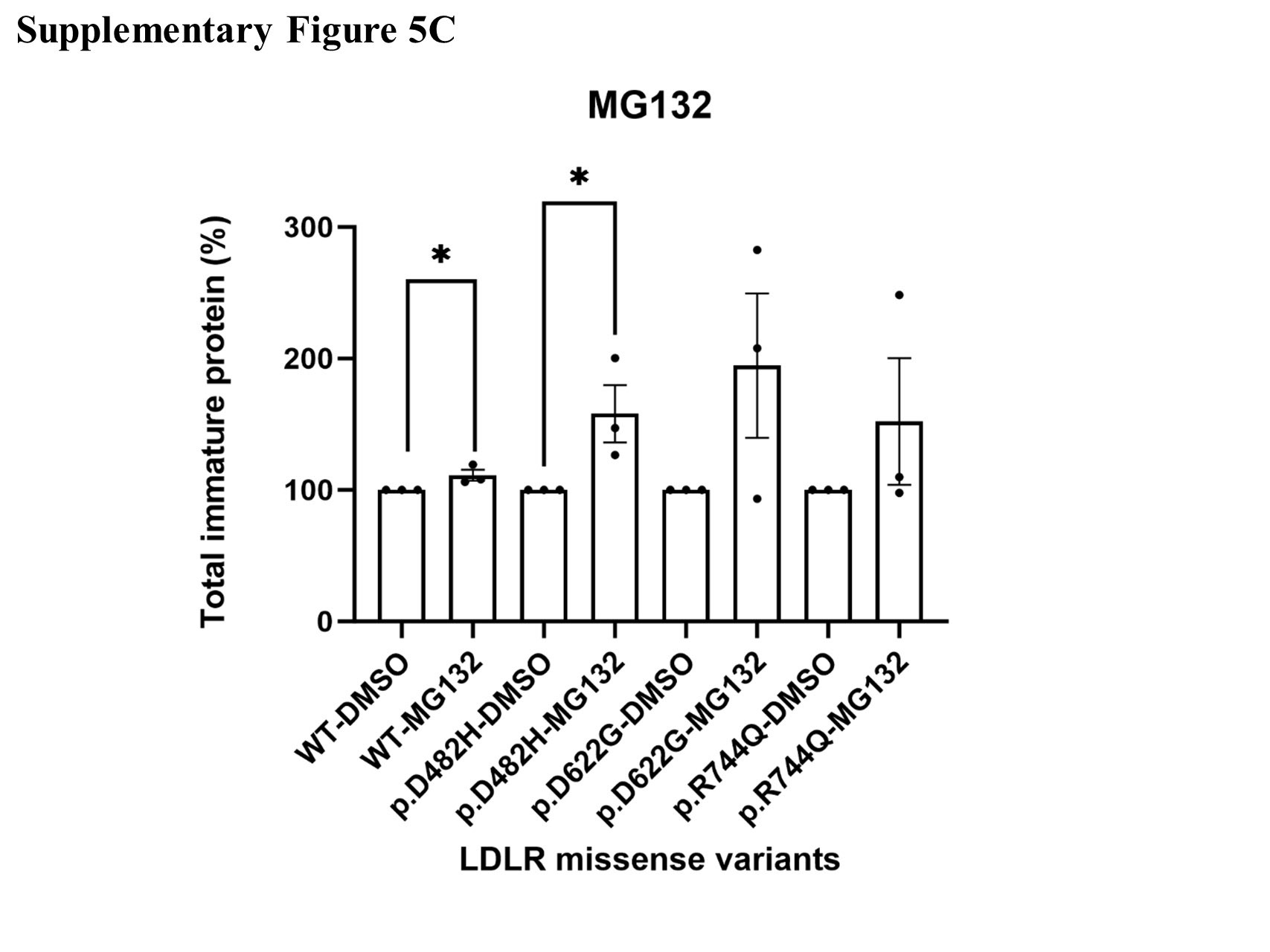

Supplement: Supplementary file 5 [file Image7.JPEG]

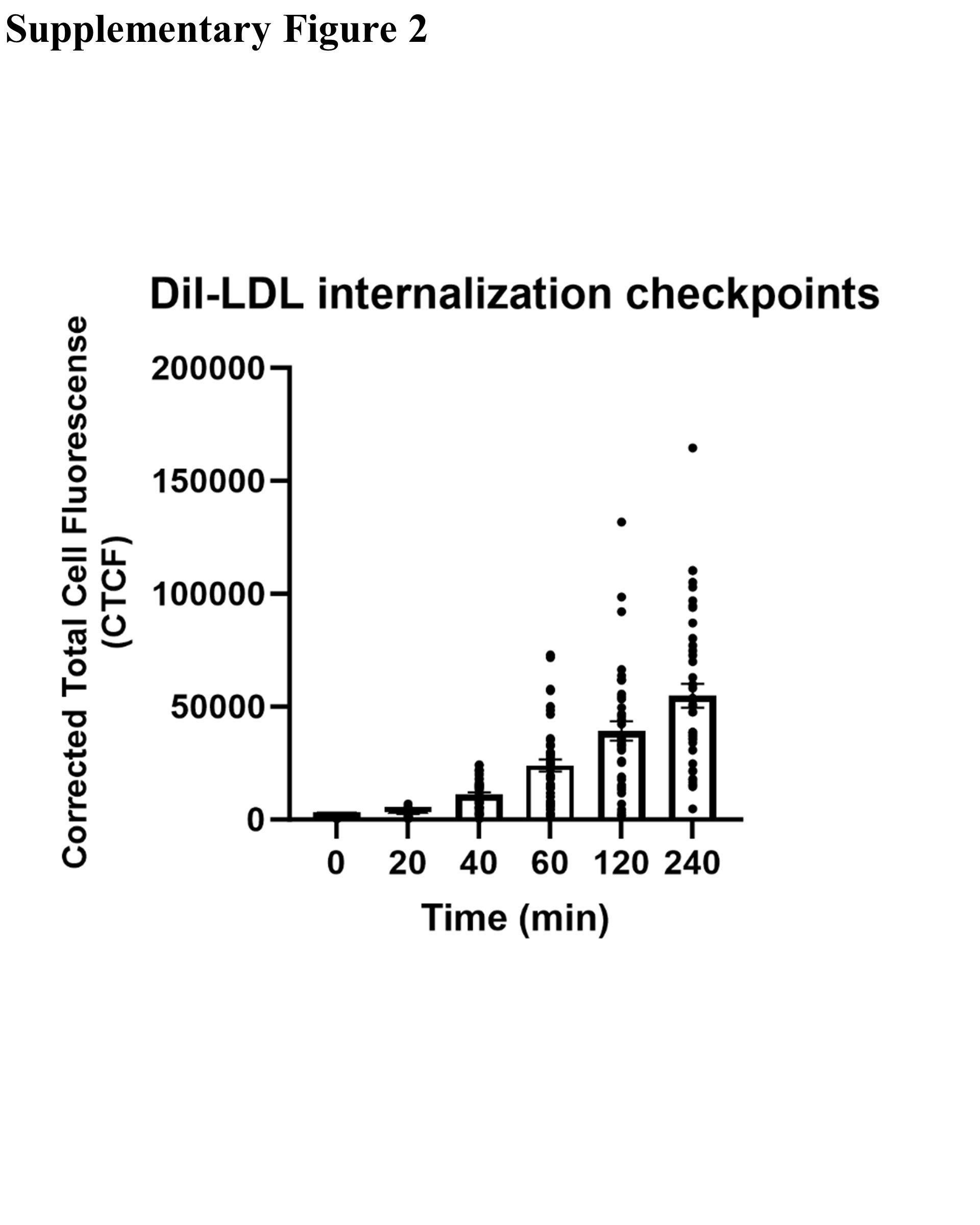

Supplement: Supplementary file 6 [file Image2.JPEG]

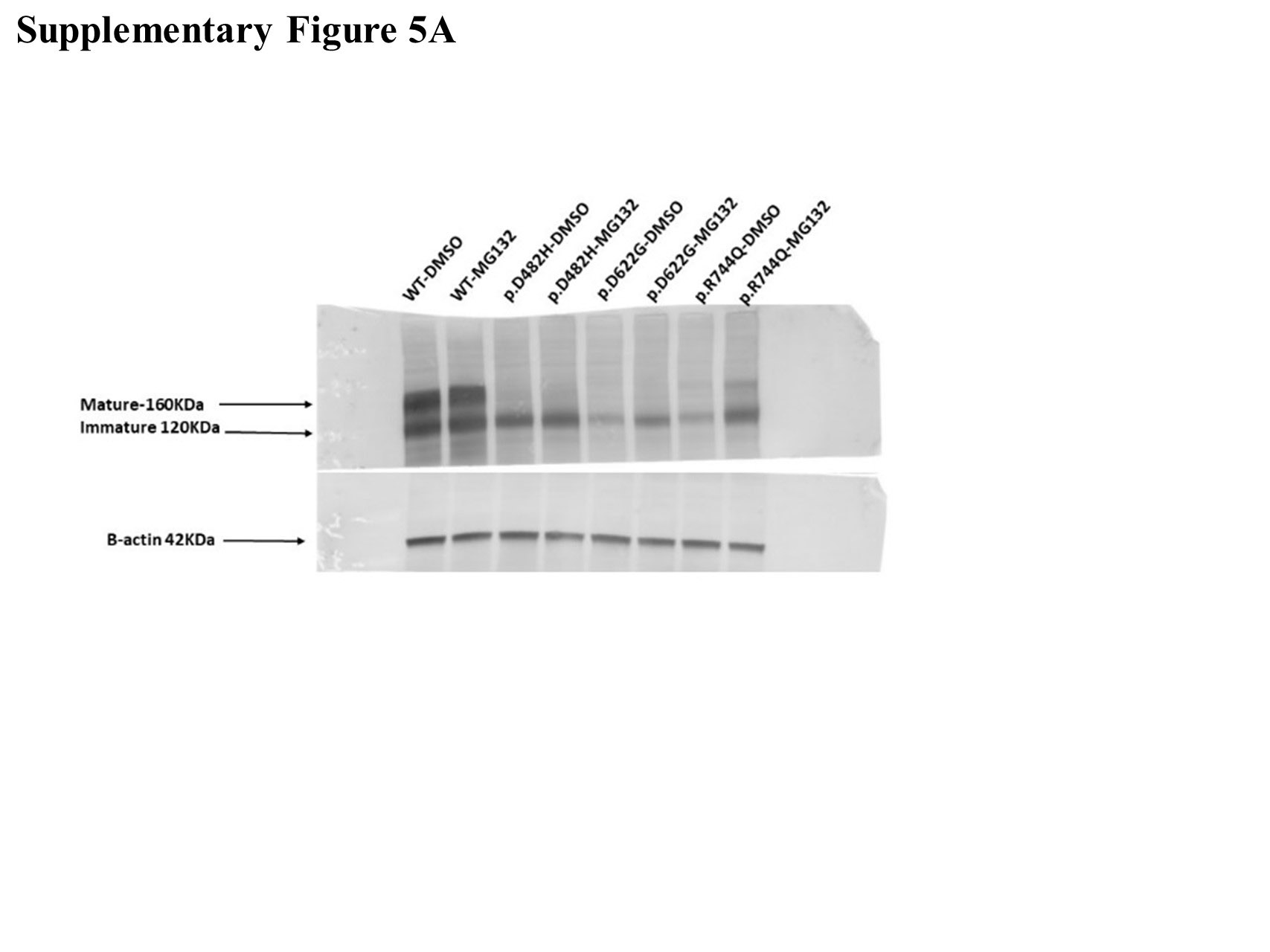

Supplement: Supplementary file 7 [file Image5.JPEG]

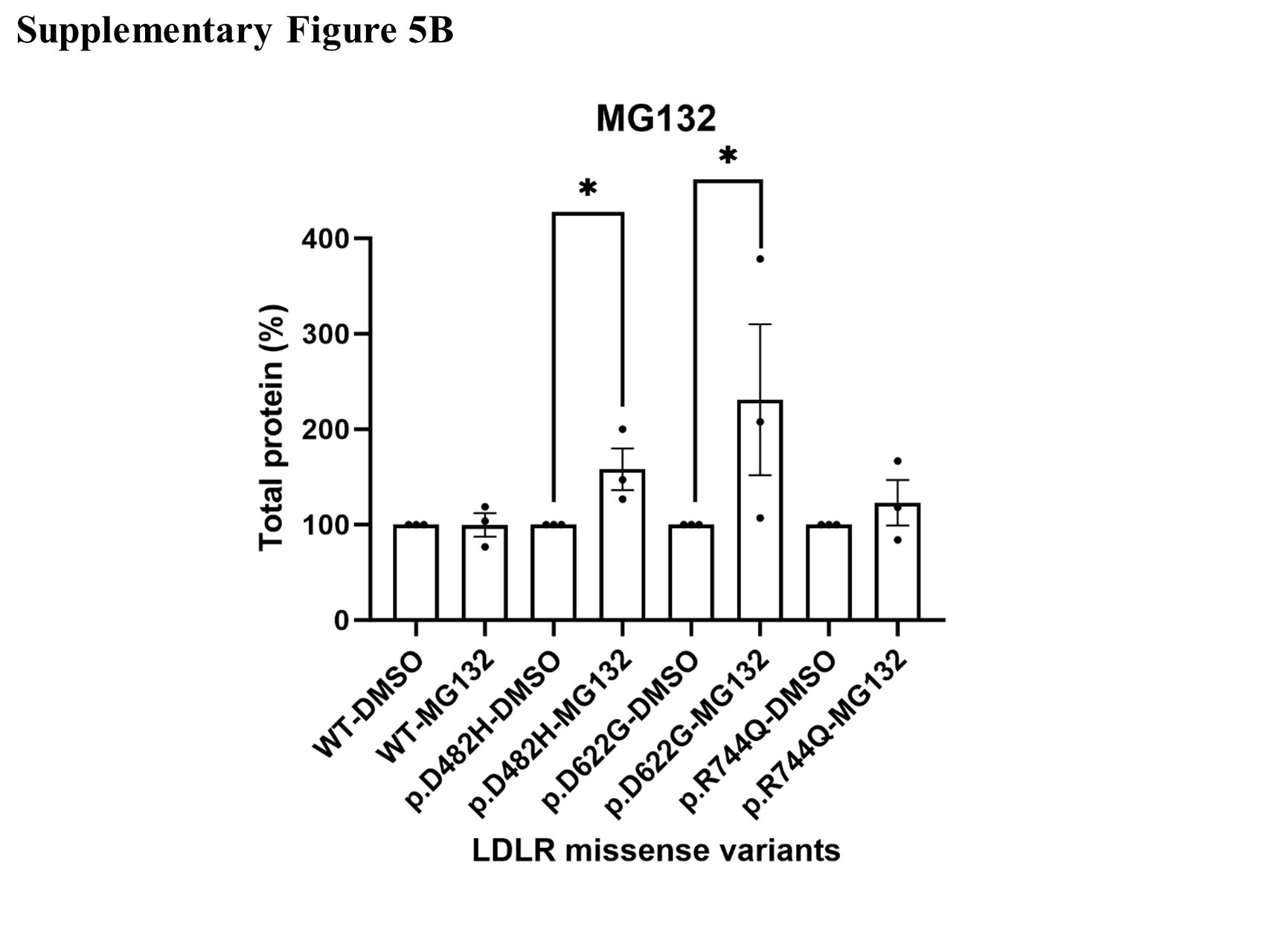

Supplement: Supplementary file 9 [file Image6.JPEG]
