# Supplementary material for: Cellular and functional evaluation of LDLR missense variants reported in hypercholesterolemic patients demonstrates their hypomorphic impacts on trafficking and LDL internalization
Source: Front Cell Dev Biol. 2024 Jul 24;12:1412236. doi: 10.3389/fcell.2024.1412236 (PMC11303217; doi:10.3389/fcell.2024.1412236)
Supplement: Supplementary file 8 [file DataSheet1.PDF]

Missense variant	SIFT	PolyPhen-2	ClinPred	Mutation Assessor	Mutation Taster	PROVEAN	Clinical significance
**p.C167F**	D (0)	PD (1)	D	H	D	D	-
**p.D178N**	D (0)	PD (0.982)	D	H	D	D	likely pathogenic
**p.C243Y**	D (0)	PD (1)	D	H	D	D	-
**p.E277K**	T (0.07)	B (0.118)	T	L	D	N	benign,likely_benign,conflicting_ interpretations_of_pathogenicity
**p.G314R**	T (0.06)	B (0.065)	T	M	D	D	Not provided, uncertain significance
**p.H327Y**	D (0)	PD (0.998)	D	M	D	D	uncertain_significance,conflicting _interpretations_of_pathogenicity
**p.D477N**	T (0.05)	PD (0.932)	D	M	D	D	Uncertain significance
**p.D622G**	D (0)	PD (0.996)	D	H	D	D	Conflicting interpretations of pathogenicity
**p.R744Q**	T (0.59)	B (0)	T	N	N	N	Likely benign, benign/likely_benign,conflicting_ interpretations_of_pathogenicity
**p.R814Q**	T (0.36)	PSD (0.606)	T	L	D	N	Likely benign, uncertain_significance,conflicting _interpretations_of_pathogenicity

Table 1: in-silico analysis of the LDLR missense variants run on Variant Effect Predictor (VEP) by Ensemble

Legend and abbreviations: Sorting Intolerant from Tolerant (SIFT); D: Deleterious, T: Tolerated. Polymorphism Phenotyping (PolyPhen); PD: Probably damaging, B: Benign, PSD: Possibly damaging. Mutation Assessor; L: Low, M: Medium, H: High. MutationTaster: D: Disease causing, N: Polymorphism. PROVEAN; D: Deleterious, N: Neutral/ Benign.
